# Fine‐root trait variation in temperate trees follows arc‐shape pattern along deep soil profiles

**DOI:** 10.1111/nph.71263

**Published:** 2026-05-14

**Authors:** Katrin Pietig, Christoph Leuschner, Heinz Coners, Martyna M. Kotowska

**Affiliations:** ^1^ Plant Ecology and Ecosystems Research, Albrecht‐von‐Haller‐Institute for Plant Sciences University of Göttingen Göttingen 37073 Germany; ^2^ Environmental Control, Northwest German Forest Research Institute Göttingen 37079 Germany; ^3^ School of Natural Sciences Macquarie University Sydney NSW 2109 Australia

**Keywords:** ectomycorrhizal associations, excavation, fine‐root morphology, root economic space, soil nutrients, soil water content, temperate trees

## Abstract

- Roots are plants' interface with the soil, controlling access to water and nutrients. Yet, fine‐root trait variation along deep soil profiles and its functional implications remain poorly understood.
- We quantified vertical fine‐root trait variation in four temperate tree species with contrasting rooting types – beech, oak, pine and Douglas fir. Roots were excavated to 380 cm depth in German Pleistocene sandy soils. Fine‐root morphological and functional traits were related to soil properties, nutrient and water availability.
- Across species, fine‐root traits followed a consistent arc‐shaped pattern with depth. Roots in the topsoil (< 50 cm) and deep subsoil (> 300 cm) exhibited acquisitive traits – high specific root length (SRL), root N content and specific root tip abundance (SRTA) indicating high metabolic activity and resource uptake potential. By contrast, roots in intermediate subsoil layers (50–300 cm) displayed more conservative traits, with thicker diameters and denser tissues. Ectomycorrhizal associations occurred throughout the soil profile.
- Our findings provide rare empirical evidence of fine‐root functional differentiation across deep soil layers, as root economic traits vary nonlinearly with depth. Thus, trees express vertically contrasting resource‐use strategies. Incorporating vertical root trait plasticity into vegetation models can improve predictions of resource uptake and carbon turnover in deep‐rooted species.

## Graphical abstract

Root trait variation along the soil depth profile in four temperate tree species.

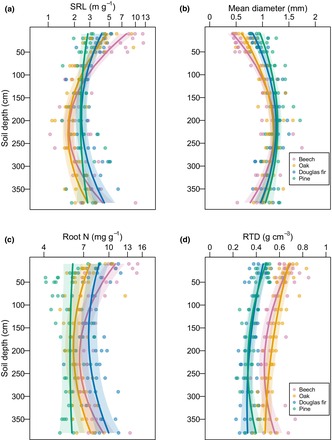

## Introduction

Soils are highly dynamic environments (Dallstream *et al*., 2025; Weemstra *et al*., 2016) often characterized by small‐scale gradients and temporal fluctuations in water and nutrient availability (Ostonen *et al*., 2007; Zhang *et al*., 2019). Tree roots, in association with their symbiotic mycorrhizal partners, are specialized for the efficient uptake and transport of these soil resources (Pregitzer *et al*., 1998; Freschet & Roumet, 2017; Meier *et al*., 2018; Kong *et al*., 2019). Roots are able to store plant‐derived carbohydrates and their turnover plays an essential role in soil biochemical cycles influencing soil carbon sequestration and soil weathering (Schenk, 2005; Fan *et al*., 2017; Weemstra *et al*., 2023; Yang *et al*., 2023). Distribution and morphology of the most distal and metabolically active components of the root network determine access to moisture and nutrients, thereby shaping plant performance and competitive advances (Freschet *et al*., 2021).

Resource uptake and retention strategies across species and environments are expected to align with the plant economic spectrum (Reich, 2014; Kramer‐Walter *et al*., 2016; Freschet *et al*., 2017; Valverde‐Barrantes *et al*., 2017). Its conceptual belowground extension uses fine‐root nitrogen concentration, root tissue density (RTD), mean root diameter and specific root length (SRL) as key traits to capture trade‐offs in resource acquisition and conservation (Bergmann *et al*., 2020; Carmona *et al*., 2021; Weigelt *et al*., 2021; Matthus *et al*., 2025). Fine‐root traits of faster return on investment associated with high N and high SRL have been related to conditions of favorable soil resource availability, while species in resource‐poor environments invest in denser, longer‐lived roots with lower N and SRL (Kramer‐Walter *et al*., 2016; Freschet *et al*., 2017; Fort & Freschet, 2020; Pierick *et al*., 2023).

This variation has also been conceptualized within the framework of multidimensional root economic space (RES; Bergmann *et al*., 2020; Matthus *et al*., 2025). Parallel to the leaf economic spectrum (Wright *et al*., 2004), one axis of the RES is organized along the fast–slow gradient of resource conservation (Kong *et al*., 2019; Bergmann *et al*., 2020). In the RES, the conservative ‘slow’ end is defined by long‐lived roots with high RTD, whereas the acquisitive ‘fast’ end is characterized by short‐lived roots with high nitrogen content (Kong *et al*., 2019; Bergmann *et al*., 2020; Matthus *et al*., 2025). A second mycorrhizal collaboration axis was identified and ranges from ‘do‐it‐yourself’ roots with high SRL values toward ‘outsourcing’ roots with thicker root cortex fraction and root diameter allowing exchange of carbohydrates vs nutrients with arbuscular mycorrhizal partners (Bergmann *et al*., 2020; Soudzilovskaia *et al*., 2020; Freschet *et al*., 2021). Although root trait data are most abundant for angiosperms, similar patterns of trait coordination consistent with the RES have also been observed in gymnosperms (Langguth *et al*., 2024). Furthermore, not all plants have arbuscular mycorrhizal associations. Many woody angiosperm and gymnosperm species exhibit predominantly ectomycorrhizal associations (ECM; Soudzilovskaia *et al*., 2025). These fungi typically colonize thinner roots with high SRL values, thereby enhancing the trees' resource acquisition capacity (Brundrett, 2009; Brundrett & Tedersoo, 2018; Bergmann *et al*., 2020; Soudzilovskaia *et al*., 2020).

Besides species‐level differences, the individual root systems of trees also show substantial variation. The remarkable plasticity in response to soil environmental variations suggests that individual plants adjust root traits along soil depth gradients in ways that reflect shifts in their functional roles (Ostonen *et al*., 2007; Germon *et al*., 2020; Zheng *et al*., 2023; Dallstream *et al*., 2025). Favorable topsoil conditions characterized by relatively high nutrient availabilities and low soil strength can support the development of highly efficient foraging root systems, given adequate soil moisture via regular precipitation inputs (Schenk, 2008; Kirfel *et al*., 2019; Maes *et al*., 2019; Zhou *et al*., 2022). As a result, the construction costs for roots in these shallow soil layers can remain low, giving a fast return on initial investment (Schenk, 2005). Traits such as high specific root length (SRL), specific root area (SRA), root nitrogen content (Root N) and a greater number of root tips enable fine roots to explore larger soil volumes and enhance the uptake of water and nutrients in the topsoil (Weemstra *et al*., 2016; Kirfel *et al*., 2019; Zhang *et al*., 2019; Bergmann *et al*., 2020; Germon *et al*., 2020; Zhou *et al*., 2022).

With limited or fluctuating topsoil water availabilities, a deeper‐reaching root system can be an effective drought‐tolerance strategy of trees (Nardini *et al*., 2016; Fan *et al*., 2017; Germon *et al*., 2020). Deep roots enable trees to access untapped soil water reservoirs and nutrient‐enriched patches at greater depths (Maeght *et al*., 2013; Brunner *et al*., 2015; Kirfel *et al*., 2019). As such, deeper soil horizons are often enriched in base cations (e.g. calcium and magnesium) derived from bedrock weathering (Kirfel *et al*., 2019; Dawson *et al*., 2020; Uhlig *et al*., 2020). Enhanced resource uptake from deeper soil layers is often facilitated by thinner and absorptive fine roots, whereas thicker fine roots of higher root branching orders are involved in the transport of the acquired water and nutrients (Germon *et al*., 2020; Freschet *et al*., 2021; Tan *et al*., 2023). Thus, variations in root morphology along deep soil profiles directly influence the capacity of trees to take up and transport resources from these deeper soil layers (Prieto *et al*., 2015; Germon *et al*., 2020).

In addition to morphological adaptations in the roots themselves, the type, extent and activity of mutualistic symbioses with mycorrhizal fungi will determine the balance between soil resource acquisition for the plant and the cost of plant‐supplied carbon to the fungus (Pena & Tibbett, 2024). Ectomycorrhizal fungi have been documented up to depths of 2 m in oak savanna and up to 4 m in bedrock fissures in various *Quercus* species in southern California (Bornyasz *et al*., 2005; Querejeta *et al*., 2007). Similarly, in Brazilian *Eucalyptus* plantations, mycorrhizal fungi have been observed at depths of 4 and 6 m (Lambais *et al*., 2017; Robin *et al*., 2019). These deep mycorrhizal associations are believed to enhance nutrient uptake and improve water availability, particularly under drought conditions (Lehto & Zwiazek, 2011; Germon *et al*., 2020). Despite these observations, there is only limited and often contradictory knowledge regarding the variation in fine‐root morphology and ectomycorrhizal associations with increasing soil depth (Prieto *et al*., 2015; Pierret *et al*., 2016; Tan *et al*., 2023; Pena & Tibbett, 2024), as most studies focus on shallower soil layers rarely extending beyond 1 m depth (Maeght *et al*., 2013; Germon *et al*., 2020).

Despite the importance of identifying general patterns in root functional traits and mycorrhizal adaptations along resource‐availability gradients, studies investigating trait variation with soil depth remain limited and often reveal inconsistent patterns (Prieto *et al*., 2015; Tückmantel *et al*., 2017; Kirfel *et al*., 2019; Zhou *et al*., 2022; Tan *et al*., 2023). To address these knowledge gaps, we conducted an excavation study to examine the plasticity of fine‐root traits and ectomycorrhizal associations along a deep vertical soil profile down to 380 cm depth. Our study included forest stands of four temperate tree species: the two deciduous broad‐leaved species European beech and Sessile oak, and the evergreen conifers Douglas fir and Scots pine. Specifically, we hypothesize the following:

1. In the topsoil, fine‐root morphological traits reflect adaptation to high resource density and localized foraging, with higher values of SRL, SRA and SRTA.
2. With increasing soil depth, we expect a shift to more conservative strategies, as subsoil layers provide fewer but more stable supply of resources, leading to larger root diameters and denser root tissues.
3. With increasing soil depth, the number of ectomycorrhizal associations declines accompanied by reduced vitality of ectomycorrhizal root tips.

## Materials and Methods

### Study site and soil properties

The studies were carried out in monocultural stands of four temperate tree species: *Fagus sylvatica* L., *Quercus petraea* (Matt.) Liebl., *Pseudotsuga menziesii* (Mirb.) Franco and *Pinus sylvestris* L. (hereafter beech, oak, Douglas fir and pine). Canopy‐closed, mature forest stands close to harvesting age were selected for the study. The coniferous tree species are usually harvested earlier due to their faster growth compared with the broad‐leaved tree species. As such, the age of the respective stands was 51 yr for Douglas fir, 80 yr for pine, 125 yr for beech and 192 yr for oak. The diameter at breast height (DBH) ranged between 33.35 cm and 53.70 cm for the coniferous species and between 42.61 and 70.00 cm for the broad‐leaved species. The study was conducted near Unterlüss in the Lüneburg Heath region, north‐western Germany (52°84′N, 10°51′E), at an elevation of 95 m above sea level. The climate in this region is classified as cool temperate, with an average annual precipitation of 818.9 mm and a mean annual temperature of 9.2°C. Daily precipitation data were obtained from the nearby weather station at Fallingbostel of the German Weather Service (DWD).

The geological substrate was formed from Pleistocene fluvioglacial sands deposited during the last Saalian glaciation (Kappler *et al*., 2019; Ehlers, 2020). For each species, three pits were excavated across 16 soil depth layers (including the organic layer), resulting in a total of 12 pits. For soil analyses, 15 soil depth layers were examined, ranging from the mineral topsoil (10–20 cm) to the very deep subsoil (350–380 cm; Table 1). The soil samples were analyzed for soil particle size distribution following the routine described in DIN ISO 11277: 2002–08 (2002). Soil textures varied with depth, with sand content ranging from 76.5% to 96.8% from the upper to deeper layers, while clay and silt contents decreased from 19.8% and 3.7% to 2.8% and 0.4%, respectively (Table 1). In particular, the beech stand exhibited slightly higher clay contents compared with the other investigated species. The soil is characterized as acidic, with a pH (H_2_O) ranging from 4.6 to 5.1. The cation exchange capacity (CEC) averaged 21.8 μmol_c_ g^−1^ in the upper layers and decreased to 2.3 μmol_c_ g^−1^ at a depth of 380 cm. Base saturation increased from 8.2% in the upper soil to 46.2% in deeper layers. The base pool (μmol_c_ g^−1^) was calculated based on the proportion of bases relative to the CEC. Soil bulk density increased with depth, reaching a mean value of 1.57 g cm^−3^ at 110 cm (no data for deeper layers available due to safety reasons; Table 1). The organic layer across all stands varied *c*. 10 cm and was included in the sampling of roots. All soil samples were subjected to physical and chemical analyses at the Department of Plant Ecology and Ecosystems Research, University of Göttingen. Bulk density analysis was carried out by the Institute of Soil Science at the Leibniz University Hannover.

**Table 1:** Averaged soil characteristics of the four investigated forest stands (mean ± SE) along the soil profile to 380 cm depth (*n* = 12) with soil chemistry (C : N ratio, pH, cation exchange capacity (CEC) and base pool) as well as physical characteristics (particle sizes of sand, silt and clay; bulk density) and plant‐available water capacity (AWC).

Soil layer (cm)	C : N	pH_H2O_	CEC (μmol_c_ g^−1^)	Base pool (μmol_c_ g^−1^)	Particle size (%)			Bulk density (g cm^−3^)	AWC (mm dm^−1^)
					Sand	Silt	Clay		
10–20	19.0 ± 2.1	4.6 ± 0.2	21.8 ± 4.4	2.0 ± 0.7	76.5 ± 2.2	19.8 ± 1.9	3.7 ± 0.6	–	19.4 ± 0.8
20–30	17.4 ± 2.0	4.8 ± 0.1	16.0 ± 4.4	0.9 ± 0.2	78.9 ± 3.1	18.2 ± 2.7	3.0 ± 0.6	1.28 ± 0.01	16.9 ± 2.0
30–40	13.8 ± 2.2	4.9 ± 0.1	8.3 ± 1.7	0.6 ± 0.1	83.9 ± 2.9	14.0 ± 2.5	2.1 ± 0.5	1.34 ± 0.01	14.2 ± 2.0
40–50	10.5 ± 1.8	4.9 ± 0.0	6.7 ± 1.3	0.6 ± 0.1	83.3 ± 2.7	14.7 ± 2.5	2.0 ± 0.4	–	14.7 ± 1.7
50–80	11.4 ± 1.4	4.8 ± 0.1	7.0 ± 1.3	0.7 ± 0.1	87.6 ± 2.7	10.4 ± 2.4	2.1 ± 0.3	1.53 ± 0.01	11.8 ± 1.9
80–110	7.9 ± 1.3	4.8 ± 0.1	6.5 ± 1.8	0.9 ± 0.3	90.3 ± 2.1	7.9 ± 1.7	1.8 ± 0.4	1.57 ± 0.02	10.0 ± 1.8
110–140	7.2 ± 1.1	5.0 ± 0.1	7.6 ± 2.5	1.6 ± 0.6	90.8 ± 1.9	7.2 ± 1.3	2.0 ± 0.7	–	9.5 ± 1.7
140–170	4.1 ± 0.9	4.9 ± 0.0	7.4 ± 3.0	2.5 ± 1.3	92.3 ± 2.3	6.0 ± 1.7	1.7 ± 0.7	–	7.8 ± 1.9
170–200	4.8 ± 0.8	5.0 ± 0.0	5.5 ± 2.1	1.9 ± 1.1	92.9 ± 2.0	5.6 ± 1.4	1.5 ± 0.6	–	7.5 ± 1.7
200–230	3.6 ± 0.8	4.9 ± 0.1	4.4 ± 1.4	1.7 ± 0.7	93.5 ± 1.1	5.4 ± 0.9	1.2 ± 0.3	–	7.4 ± 1.2
230–260	2.7 ± 0.6	4.9 ± 0.1	5.2 ± 1.9	1.9 ± 1.0	94.4 ± 1.6	4.2 ± 1.1	1.4 ± 0.5	–	6.3 ± 1.5
260–290	3.4 ± 0.7	5.0 ± 0.1	3.3 ± 0.8	1.0 ± 0.4	94.8 ± 0.9	4.2 ± 0.9	0.9 ± 0.2	–	6.0 ± 0.9
290–320	3.0 ± 0.6	4.9 ± 0.1	2.8 ± 0.5	0.9 ± 0.2	95.4 ± 1.2	2.8 ± 1.1	0.8 ± 0.1	–	5.4 ± 1.1
320–350	2.6 ± 0.6	5.0 ± 0.1	2.3 ± 0.3	1.0 ± 0.2	96.2 ± 0.8	3.2 ± 0.8	0.6 ± 0.1	–	4.7 ± 0.7
350–380	2.4 ± 0.6	5.1 ± 0.1	2.3 ± 0.3	1.1 ± 0.2	96.8 ± 0.4	2.8 ± 0.4	0.4 ± 0.1	–	3.8 ± 0.3

Volumetric soil water content (SWC in %) was continuously monitored from June 2022 to May 2023 with frequency domain reflectometry (FDR) probes (Type Enviroscan, Sentek Pty Ltd, Stepney, SA, Australia) installed in soil profiles to 480 cm depth under beech, oak, Douglas fir and pine close to the soil pits excavated for root study. To assess plant‐available water capacity within the soil profile, soil water retention curves were generated based on the gravimetric soil fractions of sand, silt and clay using the ROSETTA pedotransfer function (Zhang & Schaap, 2017) from the R package soildb (Dylan *et al*., 2024). For the fitting procedure, a bulk density of 1.5 g cm^−3^ was assumed throughout the entire profile up to 380 cm (Table 1). The derived van Genuchten parameters (van Genuchten, 1980) were used to estimate SWC at field capacity (fc) and at the permanent wilting point (pwp). Soil matric potential values for fc (−6.3 kPa) and pwp (−1500 kPa) were selected based on the regional guide for mapping soil morphological and physical properties (Schrey, 2008). The plant‐available SWC (m^3^ m^−3^) was calculated as the difference between fc and pwp, which was then scaled according to the thickness of each soil horizon to determine the plant‐available water storage capacity (AWC, mm dm^−1^; Table 1).

### Experimental design and root sampling

For the analysis of the vertical root distribution of the four tree species, the 12 soil pits were excavated to a depth of 380 cm from late autumn 2021 to early spring 2023. The pits were positioned *c*. 2 m from the nearest tree, with at least 10 m separating the three pits within each stand. In total, 156 tons of soil were excavated and sieved for roots across the 12 pits, highlighting the extensive volume of soil analyzed in this study. In October 2021, the upper 50 cm of the soil profile was sampled in 10‐cm sections using a cutting frame (25 × 25 × 10 cm), which was placed 2.6 m from the nearest mature tree. Samples taken with the cutting frame were stored in plastic bags and maintained in a cooling chamber at 7°C until they could be processed at the Department of Plant Ecology and Ecosystems Research at the University of Göttingen. For soil depths below 50 cm, an excavator (CAT M316) was utilized to dig deeper pits (150 cm × 150 cm) in 30 cm intervals until reaching a depth of 380 cm. The excavated soil samples were immediately sieved in the field using a large automatic screener (Robotrac 22) or an automatic garden sieve (Mini Screener, FleXiever – SMO bv, Eeklo, Belgium) fitted with an 8‐mm mesh size and a self‐built funnel system. The collected root samples were then stored in plastic bags and kept in a cooling chamber at 7°C until laboratory analysis.

In the laboratory at the Department of Plant Ecology and Ecosystems Research, all samples were gently washed over a 2‐mm mesh. Root samples from the upper soil layers were examined under a stereomicroscope (Zeiss Stemi 305; Carl Zeiss Microscopy GmbH, Jena, Germany) to isolate roots of the target tree species, discarding roots from understory herbs and shrubs. To obtain reference material, root strands of each tree species were followed back to the corresponding stems. Fine roots were then assigned to species based on these reference samples and established morphological characteristics (Mrak *et al*., 2016). Using calipers, the entire root fraction from each soil layer sample was categorized into different diameter classes. Roots with a diameter below 2 mm were used for the root morphological analysis and analysis of the ectomycorrhizal fungi. For the determination of the overall fine‐root mass, root samples were dried at 70°C for a minimum of 48 h and weighed using SECURA 2102‐1S and QUINTIX124‐1S scales (Sartorius Lab Instruments GmbH & Co KG, Göttingen, Germany).

### Fine‐root morphology and ECM

After sorting and washing, three intact and representative fine‐root strands (diameter at starting point 2 mm where possible) were selected from each soil layer of every pit. Mean root diameter (mm), specific root surface area (SRA, cm^2^ g^−1^), SRL (m g^−1^), RTD (g cm^−3^) and specific root tip abundance (SRTA, n g^−1^) of the root strands were determined by scanning the root segments with a flat‐bed scanner and analyzing the images with the winrhizo software (Regent Instruments Inc., Quebec, QC, Canada). Root masses of the investigated root strands were obtained by subsequently drying samples at 70°C for at least 48 h. Root N was assessed using standard physical and chemical analysis methods and using the CN elemental analyzer (Vario EL III; Elementar, Hanau, Germany) at the Department of Plant Ecology and Ecosystems Research, University of Göttingen. Fine‐root area index (RAI, m^2^ root area m^−2^ ground area) for each soil layer was calculated by multiplying SRA (m^2^ g^−1^) with the total fine‐root mass (g m^−2^) of the corresponding soil depth.

For beech, oak, pine and Douglas fir, ectomycorrhizal associations were examined, as these tree species exhibit predominantly ectomycorrhizal associations (Soudzilovskaia *et al*., 2025). The data regarding the ectomycorrhizal associations of pine were excluded from the subsequent analysis as per initial sample protocol, only three representative fine roots per soil depth were sampled and assessed. This sampling intensity proved insufficient to obtain the minimum required number of 50 living mycorrhizal root tips per soil depth for this species. Consequently, the sampling protocol was modified for the subsequent excavations of beech, oak and Douglas fir roots. From the freshly collected fine‐root material, 10 representative fine‐root sections with a length of 2–20 cm per pit and soil depth were randomly selected, preserved in 5% glycerole solution and stored at −20°C until further processing. Following thawing, roots were segmented into 4 cm rootlets, which were distributed over a grid with 36 fields. Using a random number generator, rootlets were removed from the fields and examined under a stereomicroscope (Zeiss Stemi 305; Carl Zeiss Microscopy GmbH, Jena, Germany) to assess tip numbers for different vitality status (vital, nonvital or non‐ectomycorrhizal; Pena *et al*., 2017). The root tips of each sample were counted until a number of 100 living (vital) ectomycorrhizal root tips were reached in soil depth layers down to 110 cm; in the 110 to 380 cm layers, the root tips of each soil layer were counted till a number of 50 vital ECM root tips. Due to limited root tip availability in Douglas fir roots within the upper 110 cm, counting was adjusted to a number of 50 vital ECM root tips threshold here as well. The ectomycorrhizal colonization rate (in %) gives a measure of the infection rate of vital root tips by fungi and relates the colonization only to the fraction of vital/living fine‐root tips, omitting the nonvital ones. It was calculated as follows: number of vital ECM root tips × 100/(number of vital ECM root tips + number of vital non‐ectomycorrhizal root tips; Pena *et al*., 2017). Another measure, the proportion of vital ECM in %, relates the abundance of vital/living ECM root tips to the total number of root tips (including nonvital ones) and may better reflect the functional importance of mycorrhizal colonization in the given root strands. It was calculated as follows: number of vital ECM root tips × 100/(number of vital ECM root tips + number of vital non‐ectomycorrhizal root tips + number of nonvital root tips). Vital ECM root tips were classified morphologically, following Agerer (1987–2012; cf. Pena *et al*. (2010); www.deemy.de). Morphotype characterization accounted for variations in color, branching, hyphal structure and rhizomorphs. Representative ectomycorrhizal fungi from each morphotype were documented and photographed using a SteREO V20 stereomicroscope (Carl Zeiss Microscopy GmbH, Jena, Germany) and axio vision se64 rel. 4.9 software. After analysis, rootlets were dried at 70°C for at least 48 h.

We recognize that replication at the stand level would have been preferable, yet the extensive excavation and analysis of large soil volumes made this unfeasible. Accordingly, this study represents a first comprehensive assessment of fine‐root functional traits down to a soil depth of 3.8 m that warrants repetition across sites with contrasting soil properties to derive more general insights into the deep rooting strategies of the four temperate tree species.

### Data analysis

**Fig. 1:**
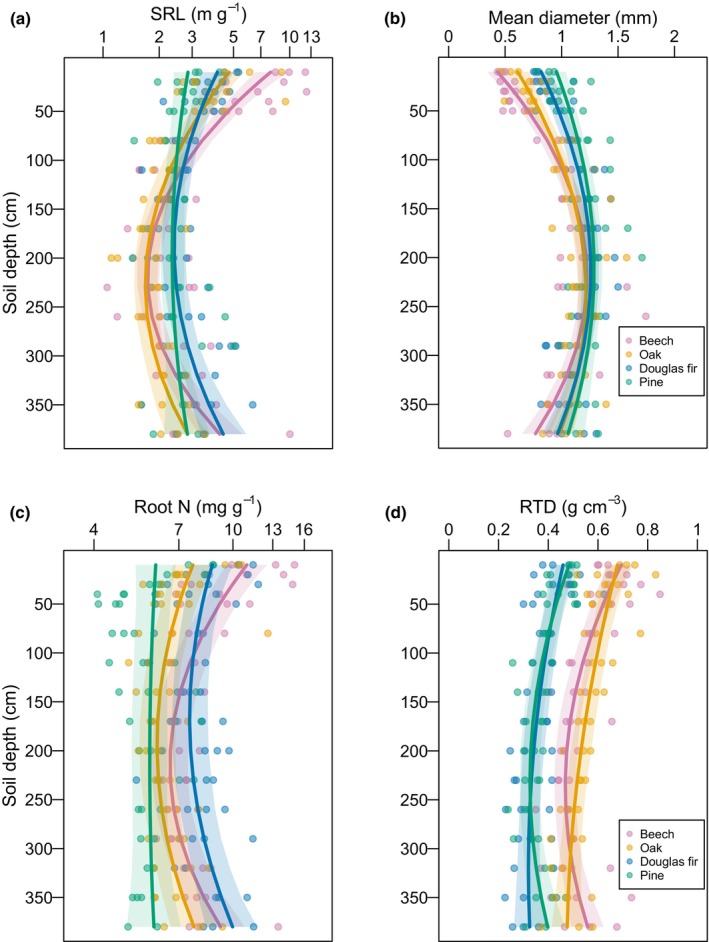
Root trait variation along the soil depth profile in beech, oak, pine and Douglas fir. Solid lines show fitted linear mixed models (LMMs) for each tree species and dots depict the observed values of the different variables (*n* = 3 soil pits per species). The response variables are (a) specific root length (SRL, m g^−1^), (b) mean diameter (mm), (c) root N concentration (mg g^−1^), and (d) root tissue density (RTD, g cm^−3^). Please note the logarithmic scale of the *x*‐axes in Fig. 1(a,c). Additional details regarding the models and their outcomes can be found in the Supporting Information.

**Fig. 2:**
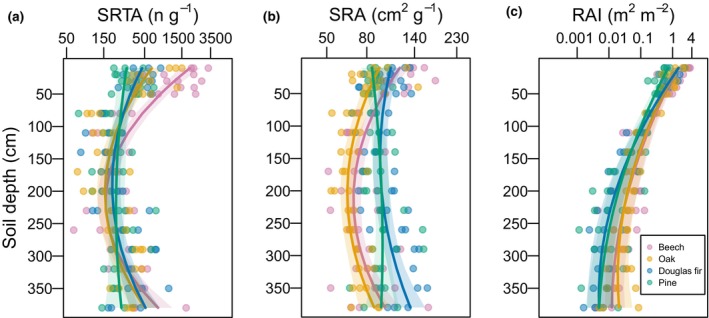
Root trait variation along the soil depth profile in in beech, oak, pine and Douglas fir. Solid lines show fitted models for each tree species and dots depict the observed values of the different variables (*n* = 3 soil pits per species). The response variables are (a) specific root tip abundance (SRTA, n g^−1^), (b) specific root area (SRA, cm^2^ g^−1^), and (c) root area index (RAI, m^2^ m^−2^). Please note the logarithmic scale of the *x*‐axes in Fig. 2 (a–c). Additional details regarding the models and their outcomes can be found in the Supporting Information.

**Fig. 3:**
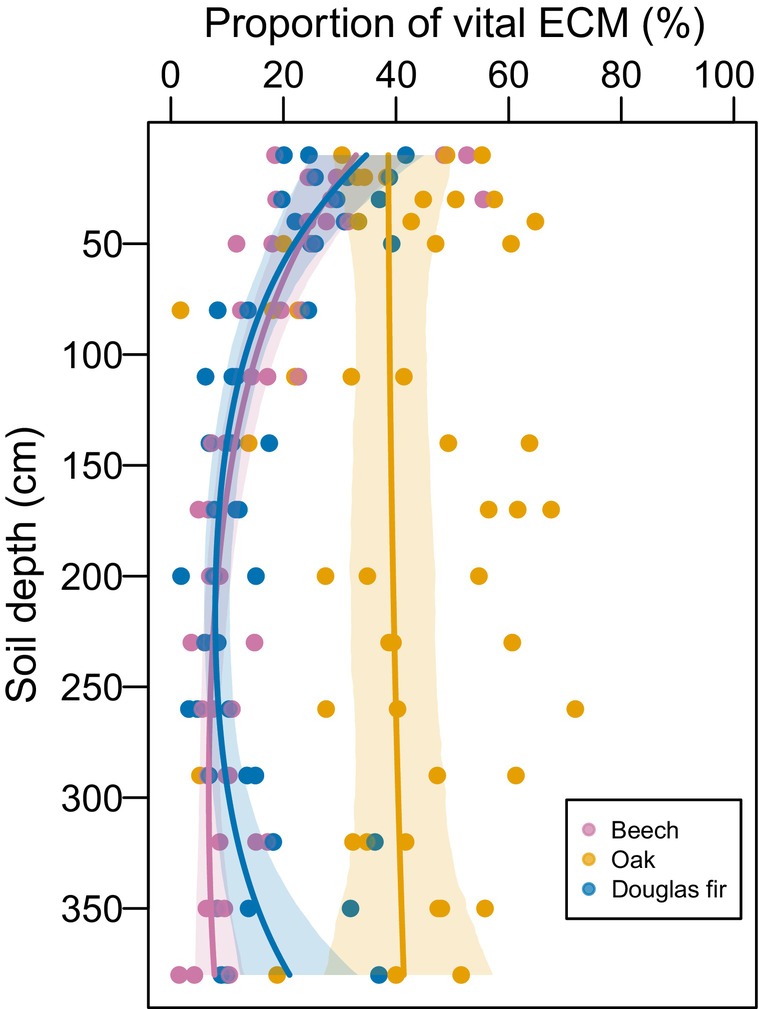
Proportion of vital ectomycorrhizal fungi (ECM; in %) as a function of species (beech, oak and Douglas fir) and soil depth. Solid lines show a generalized linear mixed model (GLMM) fitted for each tree species and dots depict the values of the proportion of vital ECM (*n* = 3 soil pits per species). Additional details regarding the model and its outcome can be found in Supporting Information Tables S16 and S17.

To test the effects of species and soil depth (and their interactions) on the target response variables (SRL, mean diameter, root N, RTD, SRA and SRTA), we employed linear mixed models (LMMs; Baayen, 2008; Figs 1, 2, 3; Supporting Information Notes S1; Tables S1–S15). For the one model, which analyzed the proportion of vital ECM root tips as the response variable, we used a two‐column matrix reflecting the counts of (1) vital ECM root tips and (2) the combined counts (sum) of nonvital and vital non‐ectomycorrhizal root tips per soil depth and applied a generalized LMM with binomial error distribution (Baayen, 2008; Fig. 3; Tables S16–S17). For ease of model convergence, we z‐transformed the predictor variable soil depth in all models and log‐transformed the response variables SRL, root N, SRA, RAI and SRTA. More information, including an overview of all employed models, their underlying assumptions, the resulting outputs and the R packages used, is provided in the Notes S1 and Tables S1–S17.

All model fitting and quantitative analyses of the plots were conducted in R 4.3.2 (R Core Team, 2024) using rstudio (RStudio Team, 2023). Data management was conducted using tidyverse (v.2.0.0; Wickham *et al*., 2019). The package ggvenn (v.0.1.10; Yan, 2023) was used to create the Venn diagram. All tables were created using Microsoft Word and Microsoft Excel.

## Results

To examine fine‐root morphological changes with increasing soil depth and across the four studied tree species, the following four traits were analyzed: SRL, mean diameter, root N and RTD (Fig. 1a–d). Across all four traits, an arc‐shaped pattern emerged with increasing soil depth, although some traits displayed this trend more prominently than others, and data variability often increased with greater soil depth. The effect of soil depth on morphological root traits also depended on the respective tree species. As such, a significant interaction between species and soil depth was observed for the following variables: SRL, mean diameter and root N (Tables S2–S27). Higher SRL values were predominantly concentrated in the upper 50 cm of the topsoil (Fig. 1a). In the subsoil (defined here as 50–290 cm), SRL values decreased sharply, with the lowest SRL values observed in most tree species at *c*. 200 cm soil depth. From the subsoil toward the deeper subsoil (defined here as 290–380 cm soil depth), SRL increased again three‐ to fourfold in oak, Douglas fir and pine, and up to 10‐fold in beech.

Root N and RTD were also higher in the topsoil than deeper in the profile (Fig. 1c,d). Both traits displayed a consistent decreasing trend to roughly half from the top‐ to the subsoil. They nearly doubled again in the deeper subsoil, where values were more variable as indicated by wider confidence intervals (CIs). By contrast, mean root diameter exhibited an inverse, arc‐shaped pattern with increasing soil depth. Smaller diameters were observed in both the topsoil and very deep subsoil, with the diameter maximum at *c*. 200 cm soil depth (Fig. 1b).

In general, the arc‐shaped trait pattern appeared to be more pronounced in the two broad‐leaved species beech and oak (Fig. 1a–d). The coniferous species often displayed a narrower range of values across morphological traits. Root N content of pine did not exhibit a clear arc‐shaped pattern (Fig. 1c).

Next to the root morphological traits of the RES, three additional morphological and functional traits – SRTA, SRA and root area index (RAI) – were investigated (Fig. 2a–c). As with other traits, a pronounced arc‐shaped pattern was also observed for these traits, which was significant for SRTA and SRA along the soil depth depending on the species (Tables S10–S13). By contrast, RAI values decreased continuously with soil depth without a marked rebound in deeper soil layers (Fig. 2c).

The highest values for SRTA, SRA and RAI were generally found in the top 50 cm of the soil (Fig. 2a–c). For SRTA, the values declined by 95–98% in the broad‐leaved species and by *c*. 90% in the conifers toward the subsoil. However, SRTA increased again in the deeper subsoil in all four species. From the subsoil to deeper subsoil, the species differences in SRTA patterns weakened, and data variability increased substantially. This pattern is different for SRA, where the two conifers exhibited consistently higher values than the broad‐leaved species along most of the soil profile except for the topsoil, where beech and oak reached the highest SRA values of all species. Moreover, while three species displayed arc‐shaped patterns for SRA, this was inversed in pine (Fig. 2b).

Beyond the morphological variation of fine roots along the soil depth gradient, this study explored ectomycorrhizal associations by quantifying the proportion of vital ectomycorrhizal fungi on all root tips (vital and nonvital) across increasing soil depths in three of the studied tree species (Fig. 3; Table S16). In all investigated tree species, ectomycorrhizal associations were present to the deepest examined soil depth of 380 cm. Along the soil profile, a clear arc‐shaped pattern in the proportion of vital ectomycorrhizal fungi was observed for Douglas fir and beech. The values for these species ranged from a minimum of 1.5% to a maximum of 56% of vital ectomycorrhizal fungi. By contrast, oak did not exhibit a distinct pattern with increasing soil depth and demonstrated considerably greater data variability (Fig. 3). The ectomycorrhizal colonization rate (%), which relates the number of vital ECMs to the total number of vital root tips (excluding nonvital root tips), ranged along the profile from 89% to 100% in Douglas fir and in beech, and was close to 100% (99% to 100%) in the profile in oak.

**Fig. 4:**
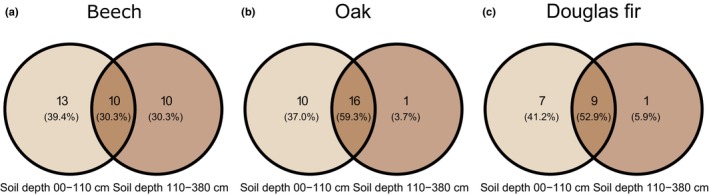
Unique and shared ectomycorrhizal morphotypes between two soil depth classes: 0–110 cm and 110–380 cm for the species beech, oak and Douglas fir. The diagrams highlight the number and proportions of ectomycorrhizal (ECM) morphotypes present in each soil depth class. Number of morphotypes is the sum of all occurring ectomycorrhizal morphotypes of the three investigated pits for the tree species.

The total number of ECM morphotypes found in the profiles decreased from beech (33) to oak (27) and finally to Douglas fir (17; Fig. 4). The Venn diagrams show that beech differed from oak and Douglas fir, with a considerable fraction of all observed morphotypes (30.3%) occurring only deeper than 110 cm depth. Approximately one‐third of the overall 33 morphotypes were unique to the upper 110 cm of soil; another third was shared between the upper and lower soil classes. In case of oak, a total of 27 morphotypes were found, with 16 morphotypes (nearly 60%) occurring in both soil depth classes. Ten morphotypes of the ectomycorrhizal fungi of oak (37%) were unique to the soil depth class of 0–110 cm, and only one morphotype was found exclusively below 110 cm. For Douglas fir, a single morphotype was unique in the deeper soil layer beyond 110 cm, while similar numbers of morphotypes occurred exclusively in the upper soil depth layers and were shared across soil depths (7 and 9 morphotypes, respectively).

**Fig. 5:**
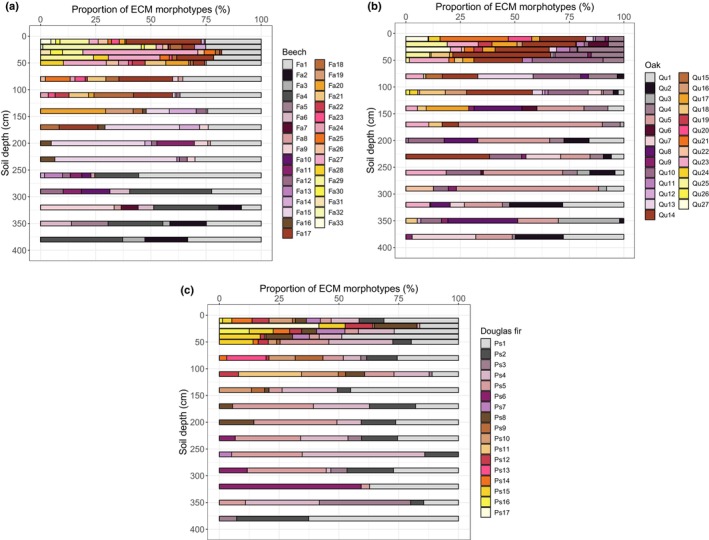
Proportion of individual ectomycorrhizal (ECM) morphotypes (% of all types found) for the different soil layers to 380 cm depth for the species (a) beech with the morphotypes Fa 1–33, (b) oak with the morphotypes Qu 1–27, and (c) Douglas fir with the morphotypes Ps 1–17. Similar colors in the three tree species do not infer similar fungal species or similar ECM morphotypes.

The analysis of the proportion of individual ECM morphotypes found at different soil depths under the three tree species revealed pronounced depth‐dependent shifts in ECM community composition (Fig. 5). While some morphotypes were consistently present throughout the entire profile and often occurred at relatively high proportions, others were restricted to the topsoil or extended only into the upper subsoil layers. By contrast, certain morphotypes appeared exclusively in the deepest subsoil layers. Some ECM morphotypes reached high proportions only in specific layers, whereas others were generally present at low proportions (Fig. 5).

**Fig. 6:**
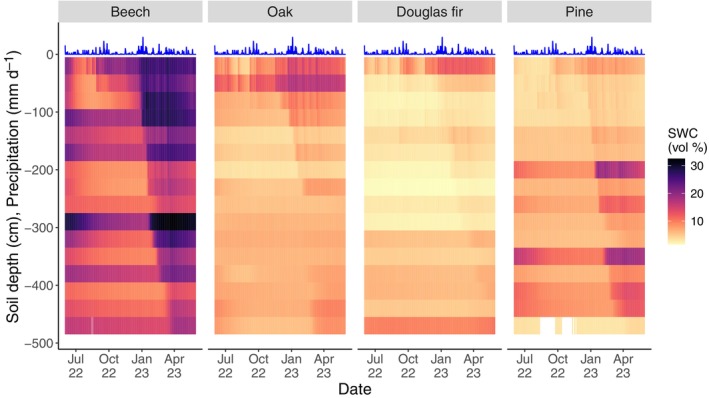
Temporal variability of volumetric soil water content (color shades) at soil depths from 20 to 470 cm, continuously monitored with frequency domain reflectometry (FDR) probes from June 2022 to May 2023 under beech, oak, Douglas fir, and pine. Precipitation data (blue bars) recorded at the Fallingbostel meteorological station are included for reference. White areas indicate data gaps.

The continuous recording of SWC in profiles to 470 cm depth in the period June 2022–May 2023 revealed a somewhat drier season in the second half of 2022 and a wetter period in the first half of the year 2023 (Fig. 6). Generally, SWC was higher under beech than under the other species. In the oak and Douglas fir stands, differences in SWC between the topsoil and the deepest subsoil layers were relatively moderate, whereas in the pine stand, SWC increased with soil depth. Generally high topsoil water contents were recorded under beech, oak and Douglas fir, but not in pine. Across all species, a slight increase in SWC was also observed between soil depths of 400 and 500 cm (Fig. 6).

## Discussion

### Functional root trait variation along the vertical profile

Along deep soil profiles down to 380 cm depth, fine roots of all four temperate species exhibited arc‐shaped patterns of trait variation. Very deep roots (300–380 cm) more closely resembled topsoil roots (0–50 cm) in all measured functional and morphological traits (SRL, SRA, SRTA, root N, mean diameter and RTD), whereas subsoil roots (50–300 cm) deviated markedly in their trait expression. Fine‐root trait variation within species thereby ranged 4–12‐fold for SRL, 2–4‐fold for mean diameter and *c*. 2–3‐fold for root N and RTD. The two investigated evergreen conifer species showed less variation along the soil profile than the deciduous broad‐leaved species.

In line with our first hypothesis, fine roots within the first 50 cm of the topsoil expressed traits related to high resource acquisition. The higher observed SRL and SRA values indicate efficient water and nutrient uptake by these more acquisitive roots, which are able to effectively explore larger soil volumes at low construction costs (Kirfel *et al*., 2019; Zhang *et al*., 2019; Fort & Freschet, 2020; Germon *et al*., 2020; Hogan *et al*., 2020; Zhou *et al*., 2022). We found that the longer and thinner topsoil fine roots were accompanied by high values of SRTA and high N contents (Fig. S1; Tables S18, S19), indicating increased physiological activity to facilitate rapid resource acquisition (Bergmann *et al*., 2020; Freschet *et al*., 2021).

The development of an efficient root foraging system can be promoted by several soil physical and chemical factors (Weemstra *et al*., 2016; Zhang *et al*., 2019; Freschet *et al*., 2021). In temperate zones, topsoil often maintains high nutrient supply rates due to a combination of low soil bulk density, high organic matter content and sufficient moisture resulting from adequate precipitation (Schrumpf *et al*., 2013; Kirfel *et al*., 2019; Maes *et al*., 2019). We found that bulk density was slightly negatively correlated with SRL, SRA, SRTA and RAI in the upper 1 m of soil (Figs S2, S3). High bulk density can limit root tip formation, whereas roots in deeper layers often overcome mechanical constraints by exploiting soil cracks or biopores formed by old root channels (Ostonen *et al*., 2007; Bengough *et al*., 2011; Weemstra *et al*., 2016; Freschet *et al*., 2021). However, the higher STRA values observed by us could also be associated with the elevated proportion of vital ECM root tips, similar to reports in other mixed temperate forests (Helmisaari *et al*., 2009; Kjøller *et al*., 2012). Higher abundance of ECM associations could also have partly contributed to the higher nitrogen content of topsoil roots, as thin fine roots with high nitrogen can result from a nitrogen‐rich ECM fungal mantle (Kong *et al*., 2019; Yan *et al*., 2022).

With regard to our second hypothesis, we expected a shift to more conservative strategies with increasing soil depth, as subsoil layers offer a lower but more stable supply of moisture and nutrients (Schenk, 2005; Germon *et al*., 2016). In these deeper layers, roots forage at lower competition intensity, but at potentially higher construction and maintenance costs, which favors thicker roots, denser root tissue and a lower SRL (Schenk, 2005; Iversen, 2010; Germon *et al*., 2020). Indeed, down to *c*. 2 m soil depth, fine‐root traits observed in this study gradually shifted toward more conservative trait expressions, as reflected by decreases in SRL, SRA, SRTA, root N and RTD. Interestingly, the trend reversed beyond 2 m depth, with trait values increasing again. We found fine roots to be thickest in the subsoil layers (50–300 cm), while roots in both the topsoil (0–50 cm) and very deep soil (300–350 cm) were markedly thinner. These unexpected patterns indicate that fine‐root trait variation aligned with our second hypothesis only partially up to a soil depth of *c*. 2 m.

Soil moisture, nutrient supply (primarily N, P, Ca, K and Mg) and bulk density are potential drivers explaining fine‐root trait variation along soil depth gradients (Ostonen *et al*., 2007; Weemstra *et al*., 2016; Zhang *et al*., 2019). Given that water and nutrients can vary at small spatial scales and do interact in soils – understanding these root–environment relationships remains challenging (Dallstream *et al*., 2025; Meyers *et al*., 2025; Tumber‐Dávila *et al*., 2022). With increasing soil depth, biological activity and mineralization rates typically decrease, leading to lower nitrogen concentrations in the subsoil (Amelung *et al*., 2018; Kirfel *et al*., 2019; Rüther *et al*., 2024). This decline in activity occurs despite our observed marked decrease in the soil C : N ratio from topsoil to subsoil, indicating a relative enrichment of nitrogen in subsoil soil organic matter (SOM; Pietig *et al*., 2026a; Fig. S4; Table S20). Root N is expected to reflect the reduced mineralization rates (Kirfel *et al*., 2019; Zhou *et al*., 2022). Contrastingly, we found a slight increase in root N content from soil depths beyond 2 m, which would support a shift toward a more acquisitive resource uptake strategy in deeper soil layers, associated with higher metabolic activity and faster resource acquisition (Bergmann *et al*., 2020; Freschet *et al*., 2021). In addition to nitrogen, other nutrients such as base cations (e.g. calcium and magnesium) may become increasingly relevant in deeper soil layers due to their potential accumulation through substrate weathering (Kolka *et al*., 1996; Kirfel *et al*., 2019). In our study, base saturation was observed to be slightly higher in both the very deep subsoil and topsoil, mirroring the vertical patterns of traits, such as SRL, SRA and RAI (except in beech). However, the overall pool of available base cations was generally low in these sandy soils (Fig. S4).

Water availability and its fluctuations affect nutrient availability through effects on ion diffusivity and mass flow of water as dependent on soil hydraulic conductance with implications for pore‐scale processes to resource redistribution across the entire soil profile (Bauke *et al*., 2022). Next to the influence of nutrient availability, fine‐root morphological and functional variations might also reflect responses to changing soil moisture regimes (Germon *et al*., 2020). At our study sites, continuously monitored SWC showed an increase specifically between 400 and 500 cm depth (Pietig *et al*., 2026a; Fig. 6). This indicates that even small‐scale changes in SWC below the deepest sampled layers may have influenced adjustments of our fine‐root traits. Higher SRL values typically are associated with an improved water absorption efficiency and thus an enhanced water uptake capacity (Tan *et al*., 2023). The annual variability of our SWC data further indicates active water uptake in these deeper soil layers across all investigated species (Fig. 6).

In general, deep soil moisture dynamics and root water uptake can differ from those in shallower layers (Schenk, 2008; Harman & Lapides, 2025). SWCs near the surface respond rapidly to individual rainfall events, whereas wet and dry phases in deeper layers tend to persist much longer and often integrate the hydrological history of water infiltration and drought from previous seasons and years (Fan *et al*., 2017; Harman & Lapides, 2025). During periods of drought, even a small number of deep fine roots can help offset the diminished water absorption by topsoil fine roots (Brinkmann *et al*., 2018; Tan *et al*., 2023). However, the drought resistance of a tree is not only determined by its species‐specific maximum rooting depth, as the water‐uptake efficiency of a tree also plays a role across different soil layers (Gessler *et al*., 2022; Kahmen *et al*., 2022; Yin *et al*., 2024). Tracer experiments for beech and Douglas fir found limited water uptake from soil layers deeper than 1 m in our study region. Even during the dry summer of 2022, both species primarily accessed water from 20 to 40 cm depth rather than the subsoil (Hackmann *et al*., 2025). While our observations of root morphological traits represent only a single snapshot in time, the continuous soil moisture monitoring across the depth profile points to a consistent rather than a flexible response of the root system to episodic soil moisture deficits. This makes it likely that the observed arc‐shaped patterns in SRL and SRTA reflect a relatively stable root morphological adjustment to the deep sandy soils at our sites, rather than being a response to certain droughts.

However, we observed a weak negative correlation between mean fine‐root diameter and the available water capacity (AWC) in the soil (Figs S2, S3). We speculate that roots in the intermediate subsoil favor a trait expression of higher hydraulic conductance serving as efficient transport pathways with lower emphasis on resource uptake (Weemstra *et al*., 2016; Freschet & Roumet, 2017; Freschet *et al*., 2021). A larger fine‐root diameter combined with a lower RTD enhances the water transport efficiency by promoting a higher hydraulic conductivity at reduced carbon cost (Wang *et al*., 2015; Bordron *et al*., 2019; Zou *et al*., 2022; Tan *et al*., 2023). Other results suggest some kind of functional differentiation of roots along deep soil gradients, whereby shallow roots mainly facilitate rapid nutrient uptake (e.g. of nitrogen) and deeper roots specialize in water uptake (Kirfel *et al*., 2017; Germon *et al*., 2020).

So far, this study is among the few that investigate fine‐root trait variation in trees along deep soil profiles. Previous research has reported inconsistent patterns in fine‐root traits with increasing soil depth (Bakker *et al*., 2008; Germon *et al*., 2016; Germon *et al*., 2018; Prieto *et al*., 2015; Tückmantel *et al*., 2017). A comparable study on *Fagus sylvatica* growing on Pleistocene sandy soils also reported an arc‐shaped pattern for several fine‐root traits, with higher SRL, SRA and root tip frequencies in the topsoil (0–20 cm) and lower subsoil (110–200 cm), but lower values in the upper subsoil (20–110 cm; Kirfel *et al*., 2019).

### Species‐specific differences of root morphology along the vertical soil gradient

Across all species, the arc‐shaped pattern of root trait variation is evident throughout the deep soil profile. However, distinct species‐specific differences emerged. Fine‐root morphology can vary considerably among taxa and species due to phylogenetic constraints (Freschet *et al*., 2021; Langguth *et al*., 2024). Gymnosperms like conifers typically have fine roots with thicker root diameters, lower SRL, fewer root tips and a lower RTD compared with angiosperms (Ostonen *et al*., 2007; Ma *et al*., 2018; Hogan *et al*., 2020; Yan *et al*., 2022). Our conifers (Douglas fir and pine) also showed consistently thicker fine roots along the entire vertical soil gradient compared with our broad‐leaved deciduous species (beech and oak), which mostly displayed higher values of SRL. The species‐specific morphological differences were found to be most pronounced within the topsoil, where the higher SRL, SRTA and smaller root diameters of the broad‐leaved species indicate more acquisitive resource uptake strategies compared with the conifers. The conifers, as evergreen species, benefit from slower root growth and resource conservation both above‐ and belowground (McCormack *et al*., 2012; Förster *et al*., 2021). Along our deep soil profile, our two broad‐leaved species revealed a higher plasticity and showed a more pronounced arc‐shaped pattern of fine‐root trait variation – particularly for SRL, mean diameter, root N, SRA and SRTA. This could relate to the generally deeper rooting of oak and beech at this site compared with the conifers (Pietig *et al*., 2026b), which could enable the former species to exploit deeper resources more efficiently.

### Ectomycorrhizal associations along the vertical depth gradient

Mycorrhizal symbioses play an important role in plant resource uptake and are closely linked to root morphological traits (Weemstra *et al*., 2016; McCormack *et al*., 2017). Despite their important ecological meaning, the distribution of ECM with increasing soil depth is largely unexplored (Robin *et al*., 2019; Germon *et al*., 2020; Pena & Tibbett, 2024). So far, only a few studies have examined ECM at greater depths (Bornyasz *et al*., 2005; Lambais *et al*., 2017; Robin *et al*., 2019).

We detected ECMs with fine roots down to a depth of 380 cm in all investigated tree species (beech, oak and Douglas fir). Contrary to our third hypothesis, yet consistent with the observed morphological traits, the proportion of vital ECM root tips in beech and Douglas fir also exhibited an arc‐shaped pattern, with higher vitality in both the topsoil and the deepest soil layers. This pattern is plausible, as ECM associations are typically more abundant in thinner roots with a high density of root tips (Yan *et al*., 2022). Furthermore, topsoil usually contains higher organic matter and nutrient content, which promotes the establishment of ECM (Querejeta *et al*., 2007). In temperate forests, ectomycorrhizal fine roots serve as the main nutrient‐absorbing structure, playing a crucial role in the uptake of nitrogen and phosphorus, which are often limiting (Pena & Tibbett, 2024). In contrast to the other species, oak showed no consistent pattern across soil depths.

Between 30 and 60% of all morphotypes occurred in both the topsoil and deeper soil layers, reflecting a considerable overlap in ECM community composition across depths (Fig. 4). Nevertheless, some morphotypes were restricted to either shallow or deep layers. Overall, a higher number of morphotypes – both shared and unique – was found in the upper 110 cm of the soil, likely reflecting the greater nutrient availability in these layers (Bornyasz *et al*., 2005). The sequencing of fungal DNA could have offered a more detailed understanding of the occurring ECM species (Lang, 2008), but was not included in the present study.

Our results confirm that beech, oak and Douglas fir roots are colonized by a considerable diversity of ECM morphotypes. Generally, a high functional diversity of fungal partners is thought to support the acquisition of nitrogen by accessing different N forms and foraging for spatially dispersed N sources (Pena & Tibbett, 2024). However, whether the available N sources can be successfully exploited depends also on the physiological flexibility of the fungi within their mycelia and the mutual benefits shared with the host tree (Cairney & Burke, 1996; Pena & Polle, 2014; Pena & Tibbett, 2024). The presence or absence of specific ECM taxa can be influenced by multiple factors. Some taxa exhibit adaptations to abiotic stress, activating nitrogen uptake only when the host experiences stress (Pena & Tibbett, 2024), while the occurrence of others is more strongly shaped by the site‐specific water regimes (Brunner *et al*., 2015; Germon *et al*., 2020). We interpret the observed vertical change in ECM morphotypes as a response to the distinct physical and chemical conditions of individual soil layers. By revealing a high ectomycorrhizal colonization rate (89–100%) to deep subsoil, our findings underpin the crucial role of mycorrhizas for tree nutrition, which likely takes place in the upper soil layers but points also to a potential indirect role in facilitating access to deeper soil water (Bornyasz *et al*., 2005; Allen, 2007; Querejeta *et al*., 2007; Lehto & Zwiazek, 2011; Robin *et al*., 2019).

### Conclusion

Our study provides rare empirical evidence of fine‐root trait variation beyond 1 m soil depth. The excavation‐based assessment revealed consistent arc‐shaped patterns of fine‐root traits in beech, oak, pine and Douglas fir growing on deep sandy soils. The observed variation demonstrates that individual trees express contrasting resource‐use strategies along vertical soil gradients, with acquisitive traits characterizing topsoil roots (< 50 cm) and very deep subsoil roots (> 300 cm), and more conservative traits in intermediate subsoil layers (50–300 cm). These findings highlight that root economics vary nonlinearly with soil depth and suggest that fine‐root trait‐environment relationships cannot yet be accurately inferred from measuring surface roots alone. Quantifying water and nutrient uptake rates will be essential for linking root traits to function, while also accounting for the role of mycorrhizal fungi. Future research should further investigate the role of deep roots in resource acquisition under drought conditions and extend studies across diverse soil types and species. Incorporating vertical root trait plasticity into models can thus improve predictions of water and nutrient uptake, carbon allocation and turnover – particularly for deep‐rooted species – and will enhance estimates of carbon storage in vegetation models.

## Competing interests

None declared.

## Author contributions

KP, CL, HC and MK planned and designed the research. KP and HC performed research. CL and MK provided funding. KP and HC analyzed data. KP and MK wrote the manuscript. All authors edited the manuscript.

## Disclaimer

The New Phytologist Foundation remains neutral with regard to jurisdictional claims in maps and in any institutional affiliations.

## Supporting information


**Fig. S1** Principal component analyses (PCA) visualizing root trait variation across soil depth.
**Fig. S2** Pearson correlation matrix with trends for the evaluated root and environmental traits.
**Fig. S3** Pearson correlation matrix with trends for the evaluated root and environmental traits.
**Fig. S4** Principal component analyses (PCA) visualizing variation of soil properties.
**Notes S1** Background information on the (G)LMM analyses.
**Table S1** Overview of models fitted.
**Table S2** Estimates of the fixed effects part of the LMM with SRL as the response variable.
**Table S3** Estimates of the random effects part of the LMM with SRL as the response variable.
**Table S4** Estimates of the fixed effects part of the LMM with mean diameter as the response variable.
**Table S5** Estimates of the random effects part of the LMM with mean diameter as the response variable.
**Table S6** Estimates of the fixed effects part of the LMM with root N as the response variable.
**Table S7** Estimates of the random effects part of the LMM with root N as the response variable.
**Table S8** Estimates of the fixed effects part of the LMM with RTD as the response variable.
**Table S9** Estimates of the random effects part of the LMM with RTD as the response variable.
**Table S10** Estimates of the fixed effects part of the LMM with SRTA as the response variable.
**Table S11** Estimates of the random effects part of the LMM with SRTA as the response variable.
**Table S12** Estimates of the fixed effects part of the LMM with SRA as the response variable.
**Table S13** Estimates of the random effects part of the LMM with SRA as the response variable.
**Table S14** Estimates of the fixed effects part of the LMM with RAI as the response variable.
**Table S15** Estimates of the random effects part of the LMM with RAI as the response variable.
**Table S16** Estimates of the fixed effects part of the GLMM with proportion of vital ECM as the response variable.
**Table S17** Estimates of the random effects part of the GLMM with proportion of vital ECM as the response variable.
**Table S18** Trait loadings of the PCA of the root economic traits; SRL, Mean Dia., root N and RTD.
**Table S19** Trait loadings of the PCA of the root traits; SRL, Mean Dia., root N, RTD, SRA and SRTA.
**Table S20** Trait loadings of the PCA of the soil properties.
Please note: Wiley is not responsible for the content or functionality of any Supporting Information supplied by the authors. Any queries (other than missing material) should be directed to the *New Phytologist* Central Office.

## Data Availability

The data that support the findings of this study are openly available in GRO.data at doi: 10.25625/RQWJ83 (reference no.: RQWJ83).
