# Supplementary material for: Fine‐root trait variation in temperate trees follows arc‐shape pattern along deep soil profiles
Source: New Phytol. 2026 May 14;251(3):1101–15. doi: 10.1111/nph.71263 (PMC13326498; doi:10.1111/nph.71263)
Supplement: Supplementary file 1 — Fig. S1 Principal component analyses (PCA) visualizing root trait variation across soil depth. Fig. S2 Pearson correlation matrix with trends for the evaluated root and environmental traits. Fig. S3 Pearson correlation matrix with trends for the evaluated root and environmental traits. Fig. S4 Principal component analyses (PCA) visualizing variation of soil properties. Notes S1 Background information on the (G)LMM analyses. Table S1 Overview of models fitted. Table S2 Estimates of the fixed effects part of the LMM with SRL as the response variable. Table S3 Estimates of the random effects part of the LMM with SRL as the response variable. Table S4 Estimates of the fixed effects part of the LMM with mean diameter as the response variable. Table S5 Estimates of the random effects part of the LMM with mean diameter as the response variable. Table S6 Estimates of the fixed effects part of the LMM with root N as the response variable. Table S7 Estimates of the random effects part of the LMM with root N as the response variable. Table S8 Estimates of the fixed effects part of the LMM with RTD as the response variable. Table S9 Estimates of the random effects part of the LMM with RTD as the response variable. Table S10 Estimates of the fixed effects part of the LMM with SRTA as the response variable. Table S11 Estimates of the random effects part of the LMM with SRTA as the response variable. Table S12 Estimates of the fixed effects part of the LMM with SRA as the response variable. Table S13 Estimates of the random effects part of the LMM with SRA as the response variable. Table S14 Estimates of the fixed effects part of the LMM with RAI as the response variable. Table S15 Estimates of the random effects part of the LMM with RAI as the response variable. Table S16 Estimates of the fixed effects part of the GLMM with proportion of vital ECM as the response variable. Table S17 Estimates of the random effects part of the GLMM with proportion of vital ECM as the response vari [file NPH-251-1101-s001.pdf]

New Phytologist

***New Phytologist* Supporting Information**

Article title: Fine-root trait variation in temperate trees follows arc-shape pattern along deep soil profiles
Authors: Katrin Pietig, Christoph Leuschner, Heinz Coners, Martyna M. Kotowska
Article acceptance date: 23 April 2026

The following Supporting Information is available for this article:

Notes S1 Background Information on the (G)LMM analyses.

Table S1 Overview of models fitted.

Table S2 Estimates of the fixed effects part of the LMM with SRL as the response variable.

Table S3 Estimates of the random effects part of the LMM with SRL as the response variable.

Table S4 Estimates of the fixed effects part of the LMM with mean diameter as the response variable.

Table S5 Estimates of the random effects part of the LMM with mean diameter as the response variable.

Table S6 Estimates of the fixed effects part of the LMM with root N as the response variable.

Table S7 Estimates of the random effects part of the LMM with root N as the response variable.

Table S8 Estimates of the fixed effects part of the LMM with RTD as the response variable.

Table S9 Estimates of the random effects part of the LMM with RTD as the response variable.

Table S10 Estimates of the fixed effects part of the LMM with SRTA as the response variable.

Table S11 Estimates of the random effects part of the LMM with SRTA as the response variable.

Table S12 Estimates of the fixed effects part of the LMM with SRA as the response variable.

Table S13 Estimates of the random effects part of the LMM with SRA as the response variable.

Table S14 Estimates of the fixed effects part of the LMM with RAI as the response variable.

Table S15 Estimates of the random effects part of the LMM with RAI as the response variable.

Table S16 Estimates of the fixed effects part of the GLMM with proportion of vital ECM as the response variable.

Table S17 Estimates of the random effects part of the GLMM with proportion of vital ECM as the response variable.

Fig. S1 Principal component analyses (PCA) visualizing root trait variation across soil depth.

Table S18 Trait loadings of the PCA of the root economic traits; SRL, Mean Dia., root N and RTD.

Table S19 Trait loadings of the PCA of the root traits; SRL, Mean Dia., root N, RTD, SRA and SRTA.

Fig. S2 Pearson correlation matrix with trends for the evaluated root and environmental traits.

Fig. S3 Pearson correlation matrix with trends for the evaluated root and environmental traits.

Fig. S4 Principal component analyses (PCA) visualizing variation of soil properties.

Table S20 Trait loadings of the PCA of the soil properties.

Notes S1 Background Information on the (G)LMM analyses (Fig. 1 & Fig. 2 & Fig. 4)

To test the effects of species and soil depth (and their interactions) on the target response variables (SRL, mean diameter, root N, RTD, SRA, and SRTA), we employed Linear Mixed Models (LMMs) (Baayen, 2008) (Fig. 1, Fig. 2, Fig. 3, Table S1 - Table S15). For the one model, which analyzed the proportion of vital ECM root tips as the response variable, we used a two-column matrix reflecting the counts of vital and non-vital ECM root tips per soil depth and applied a Generalized Linear Mixed Model with binomial error distribution (Baayen 2008) (Fig. 3, Table S16 - Table S17). Table S1 represents an overview of all employed models and their underlying assumptions, while all the detailed model results are available in Table S2 - Table S17.

Fixed effects in all models included species, soil depth, soil depth squared, and their interactions, based on the hypothesis that species effects on our response variables would vary with increasing soil depth. Excavated pits were incorporated as random intercept effect, while soil depth and its squared term were modeled as random slopes (Barr et al., 2013; Schielzeth and Forstmeier, 2009) (Table S3 - Table S17). Initially, we used maximal models (Barr et al., 2013) that included parameters for correlations among random intercepts and slopes. However, these parameters were estimated near or at an absolute value of one in nearly all models, indicating non-identifiability (Matuschek et al., 2017) (Table S1). Consequently, we removed these parameters, leading to only a minor reduction in model fit. Only in the model with RAI as response, these parameters were retained to account for correlations between random intercepts and slopes.

Given the expectation that fixed effects would be significant, a full null model comparison was unnecessary. For ease of model convergence, we z-transformed the predictor variable soil depth in all models (Schielzeth, 2010) and log-transformed the response variables SRL, root N, SRA, RAI and SRTA. Model assumptions for those with a Gaussian error distribution were verified by visual inspection of residuals using QQ plots (Field, 2005) and residuals versus fitted values plots (Quinn and Keough, 2002). No indications of normality or homoscedasticity violations were observed. Collinearity was assessed in models without interaction terms and did not indicate any concerns (Quinn and Keough, 2002).

To assess model stability, we systematically excluded levels of the grouping factors one at a time (Nieuwenhuis et al., 2012). Most models displayed good stability (Table S1). For the binomial model analyzing the proportion of vital ECM, the dispersion parameter of 0.094 suggested that the model response was heavily under-dispersed. Additional stability checks revealed that 28 models for stability testing did not converge, indicating that results from the binomial model should be interpreted with caution.

Model fitting was performed in R (version 4.3.2; R Core Team, (2024)) using the lmer resp. glmer functions from the lme4 package (version 1.1-35.1) (Bates et al., 2015). Variance Inflation Factors were calculated using the vif function from the car package (version 3.1-2) (Fox and Weisberg, 2011). Model stability was assessed and model estimates were bootstrapped using the bootMer function from lme4. Analyses were based on 188 observations for SRL, mean diameter, root N, SRA, RAI, RTD, and SRTA measured from 12 pits. In the binomial model evaluating the proportion of vital ECM, 143 observations were obtained from 9 pits.

Table S1 Overview of models fitted.

Model	Response	Transformation of response	Error structure	Correlations included	Model stability
Fig. 1a; Table S2; Table S3	SRL	log	Gaussian	No	Moderate
Fig. 1b; Table S4; Table S5	Mean Dia.	-	Gaussian	No	Good
Fig. 1c; Table S6; Table S7	Root N	log	Gaussian	No	Good
Fig. 1d; Table S8; Table S9	RTD	-	Gaussian	No	Good
Fig. 2a; Table S10; Table S11	SRTA	log	Gaussian	No	Good
Fig. 2b; Table S12; Table S13	SRA	log	Gaussian	No	Good
Fig. 2c; Table S14; Table S15	RAI	log	Gaussian	Yes	Moderate
Fig. 3; Table S16; Table S17	Prop. of vital ECM	-	Binomial	No	Good

Table S2 Estimates of the fixed effects part of the LMM with SRL as the response variable (estimates and standard errors, together with confidence limits, significance tests, and the range of estimates obtained when dropping levels of grouping factors one at a time).

	Estimate	Std. error	CL_{lower}	CL_{upper}	F	df	P	min	max
(Intercept)	0.649	0.072	0.511	0.793				0.547	0.748
speciesdouglasfir [(1)]	0.237	0.101	0.038	0.436				0.039	0.439
speciesoak	-0.046	0.101	-0.245	0.147				-0.096	-0.020
speciespine	0.209	0.102	-0.008	0.413				0.070	0.365
soildepth [(2)]	-0.453	0.048	-0.549	-0.363				-0.498	-0.402
I(soildepth^2)	0.507	0.058	0.384	0.621				0.462	0.575
speciesdouglasfir: soildepth	0.357	0.068	0.230	0.486				0.246	0.435
speciesoak: soildepth	0.127	0.067	-0.001	0.256				0.054	0.191
speciespine: soildepth	0.411	0.068	0.277	0.554				0.338	0.449
speciesdouglasfir: I(soildepth^2)	-0.263	0.079	-0.423	-0.111	8.951	3, 168	<0.001	-0.374	-0.181
speciesoak: I(soildepth^2)	-0.183	0.079	-0.337	-0.020				-0.194	-0.166
speciespine: I(soildepth^2)	-0.425	0.081	-0.574	-0.262				-0.499	-0.363

[(1)] species was dummy coded with beech being the reference level; the indicated significance test refers to the overall effect of species

[(2)] z-transformed to a mean of zero and a standard deviation (sd) of 1; mean and sd of the original variable were 167.5 and 121.8 cm, respectively.

Table S3 Estimates of the random effects part of the LMM with SRL as the response variable.

Groups	Effect [(1)]	Std.Dev.
pit	(Intercept)	<0.001
	soil depth	<0.001
	I(soil depth^2)	0.028

[(1)] '(Intercept)' denotes a random intercept effect; others indicate random slopes effect. Species was dummy coded with beech being the reference level and then centered. Soil depth was z-transformed to a mean of 0 and a standard deviation of 1; mean and sd of the original variables were 167.5 and 121.8 cm.

Table S4 Estimates of the fixed effects part of the LMM with mean diameter as the response variable (estimates and standard errors, together with confidence limits, significance tests, and the range of estimates obtained when dropping levels of grouping factors one at a time).

	Estimate	Std. error	CL_{lower}	CL_{upper}	F	df	P	min	max
(Intercept)	1.177	0.037	1.104	1.246				1.105	1.246
speciesoak [(1)]	-0.016	0.050	-0.121	0.085				-0.035	0.014
speciesdouglasfir	0.063	0.050	-0.037	0.172				-0.011	0.186
speciespine	0.099	0.051	-0.011	0.199				0.034	0.154
soildepth [(2)]	0.239	0.024	0.189	0.289				0.218	0.251
I(soildepth^2)	-0.263	0.028	-0.317	-0.208				-0.297	-0.232
speciesoak: soildepth	-0.034	0.033	-0.1	0.037				-0.056	-0.007
speciesdouglasfir: soildepth	-0.115	0.034	-0.179	-0.043				-0.144	-0.069
speciespine: soildepth	-0.147	0.034	-0.234	-0.098				-0.178	-0.106
speciesoak: I(soildepth^2)	0.092	0.039	0.016	0.171	4.181	3, 30	< 0.05	0.075	0.104
speciesdouglasfir: I(soildepth^2)	0.107	0.039	0.023	0.185				0.027	0.162
speciespine: I(soildepth^2)	0.143	0.040	0.046	0.214				0.107	0.173

[(1)] species was dummy coded with beech being the reference level; the indicated test refers to the overall effect of species

[(2)] z-transformed to a mean of zero and a standard deviation (sd) of 1; mean and sd of the original variable were 167.5 and 121.8 cm, respectively.

Table S5 Estimates of the random effects part of the LMM with mean diameter as the response variable.

Groups	Effect [(1)]	Std.Dev.
pit	(Intercept)	0.018
	soil depth	<0.001
	I(soil depth^2)	<0.001

[(1)] '(Intercept)' denotes a random intercept effect; others indicate random slopes effect. Species was dummy coded with beech being the reference level and then centered. Soil depth was z-transformed to a mean of 0 and a standard deviation of 1; mean and sd of the original variables were 167.5 and 121.8 cm.

Table S6 Estimates of the fixed effects part of the LMM with root N as the response variable (estimates and standard errors, together with confidence limits, significance tests, and the range of estimates obtained when dropping levels of grouping factors one at a time).

	Estimate	Std. error	CL_{lower}	CL_{upper}	F	df	P	min	max
(Intercept)	1.916	0.060	1.791	2.030				1.900	1.945
speciesoak [(1)]	-0.109	0.085	-0.276	0.065				-0.137	-0.093
speciesdouglasfir	0.100	0.085	-0.073	0.281				0.022	0.152
speciespine	-0.160	0.086	-0.326	0.015				-0.230	-0.093
soildepth [(2)]	-0.144	0.025	-0.191	-0.095				-0.171	-0.121
I(soildepth^2)	0.178	0.029	0.122	0.233				0.132	0.214
speciesoak: soildepth	0.096	0.035	0.023	0.166				0.068	0.124
speciesdouglasfir: soildepth	0.143	0.036	0.074	0.213				0.120	0.170
speciespine: soildepth	0.133	0.036	0.063	0.206				0.110	0.160
speciesoak: I(soildepth^2)	-0.075	0.042	-0.159	0.011	3.939	3,12	<0.05	-0.112	-0.029
speciesdouglasfir: I(soildepth^2)	-0.087	0.042	-0.165	-0.005				-0.124	-0.041
speciespine: I(soildepth^2)	-0.164	0.043	-0.253	-0.079				-0.200	-0.118

[(1)] species was dummy coded with beech being the reference level; the indicated test refers to the overall effect of species
[(2)] z-transformed to a mean of zero and a standard deviation (sd) of 1; mean and sd of the original variable were 167.5 and 121.8 cm, respectively.

Table S7 Estimates of the random effects part of the LMM with root N as the response variable.

Groups	Effect [(1)]	Std.Dev.
pit	(Intercept)	0.082
	soil depth	<0.001
	I(soil depth^2)	0.007

[(1)] '(Intercept)' denotes a random intercept effect; others indicate random slopes effect. Species was dummy coded with beech being the reference level and then centered. Soil depth was z-transformed to a mean of 0 and a standard deviation of 1; mean and sd of the original variables were 167.5 and 121.8 cm.

Table S8 Estimates of the fixed effects part of the LMM with RTD as the response variable (estimates and standard errors, together with confidence limits, significance tests, and the range of estimates obtained when dropping levels of grouping factors one at a time).

	Estimate	Std. error	CL_{lower}	CL_{upper}	F	df	P	min	max
(Intercept)	0.490	0.021	0.445	0.530				0.479	0.496
speciesoak [(1)]	0.061	0.029	0.000	0.121				0.051	0.071
speciesdouglasfir	-0.136	0.029	-0.197	-0.074				-0.168	-0.111
speciespine	-0.150	0.030	-0.211	-0.095				-0.169	-0.127
soildepth [(2)]	-0.076	0.011	-0.097	-0.054				-0.086	-0.069
I(soildepth^2)	0.064	0.012	0.040	0.089				0.056	0.077
speciesoak: soildepth	0.000	0.015	-0.029	0.031				-0.012	0.017
speciesdouglasfir: soildepth	0.021	0.015	-0.009	0.051				0.015	0.031
speciespine: soildepth	0.024	0.015	-0.004	0.054				0.018	0.034
speciesoak: I(soildepth^2)	-0.046	0.017	-0.079	-0.011	2.835	3,11	0.087	-0.059	-0.037
speciesdouglasfir: I(soildepth^2)	-0.043	0.017	-0.077	-0.009				-0.056	-0.033
speciespine: I(soildepth^2)	-0.017	0.017	-0.051	0.016				-0.030	-0.007

[(1)] species was dummy coded with beech being the reference level; the indicated test refers to the overall effect of species
[(2)] z-transformed to a mean of zero and a standard deviation (sd) of 1; mean and sd of the original variable were 167.5 and 121.8 cm, respectively.

Table S9 Estimates of the random effects part of the LMM with RTD as the response variable.

Groups	Effect [(1)]	Std.Dev.
pit	(Intercept)	0.026
	soil depth	0.008
	I(soil depth^2)	0.007

[(1)] '(Intercept)' denotes a random intercept effect; others indicate random slopes effect. Species was dummy coded with beech being the reference level and then centered. Soil depth was z-transformed to a mean of 0 and a standard deviation of 1; mean and sd of the original variables were 167.5 and 121.8 cm.

Table S10 Estimates of the fixed effects part of the LMM with SRTA as the response variable (estimates and standard errors, together with confidence limits, significance tests, and the range of estimates obtained when dropping levels of grouping factors one at a time).

	Estimate	Std. error	CL_{lower}	CL_{upper}	F	df	P	min	max
(Intercept)	5.213	0.123	4.980	5.448				5.035	5.352
speciesoak [1]	-0.114	0.174	-0.455	0.225				-0.254	0.064
speciesdouglasfir	0.061	0.174	-0.308	0.383				-0.095	0.239
speciespine	0.182	0.175	-0.184	0.509				0.043	0.360
soildepth [2]	-0.726	0.082	-0.876	-0.578				-0.762	-0.675
I(soildepth^2)	0.854	0.096	0.668	1.028				0.798	0.938
speciesoak: soildepth	0.391	0.116	0.176	0.606				0.340	0.427
speciesdouglasfir: soildepth	0.563	0.116	0.334	0.784				0.485	0.640
speciespine: soildepth	0.627	0.117	0.394	0.852				0.577	0.663
speciesoak: I(soildepth^2)	-0.307	0.135	-0.558	-0.039	9.458	3,26	<0.001	-0.397	-0.219
speciesdouglasfir: I(soildepth^2)	-0.454	0.135	-0.706	-0.181				-0.544	-0.283
speciespine: I(soildepth^2)	-0.756	0.139	-1.024	-0.496				-0.839	-0.698

(1) species was dummy coded with beech being the reference level; the indicated test refers to the overall effect of species

(2) z-transformed to a mean of zero and a standard deviation (sd) of 1; mean and sd of the original variable were 167.5 and 121.8 cm, respectively.

Table S11 Estimates of the random effects part of the LMM with SRTA as the response variable.

Groups	Effect [1]	Std.Dev.
pit	(Intercept)	<0.001
	soil depth	<0.001
	I(soil depth^2)	<0.001

(1) '(Intercept)' denotes a random intercept effect; others indicate random slopes effect. Species was dummy coded with beech being the reference level and then centered. Soil depth was z-transformed to a mean of 0 and a standard deviation of 1; mean and sd of the original variables were 167.5 and 121.8 cm.

Table S12 Estimates of the fixed effects part of the LMM with SRA as the response variable (estimates and standard errors, together with confidence limits, significance tests, and the range of estimates obtained when dropping levels of grouping factors one at a time).

	Estimate	Std. error	CL_{lower}	CL_{upper}	F	df	P	min	max
(Intercept)	4.255	0.044	4.161	4.344				4.215	4.297
speciesoak [(1)]	-0.087	0.062	-0.22	0.048				-0.096	-0.072
speciesdouglasfir	0.285	0.062	0.162	0.414				0.183	0.420
speciespine	0.289	0.063	0.159	0.402				0.192	0.383
soildepth [(2)]	-0.153	0.030	-0.214	-0.09				-0.183	-0.120
I(soildepth^2)	0.194	0.037	0.119	0.27				0.160	0.231
speciesoak: soildepth	0.059	0.042	-0.028	0.149				0.019	0.111
speciesdouglasfir: soildepth	0.180	0.042	0.095	0.264				0.109	0.228
speciespine: soildepth	0.200	0.042	0.087	0.255				0.161	0.223
speciesoak: I(soildepth^2)	-0.046	0.049	-0.143	0.056	6.975	3,162	<0.001	-0.059	-0.021
speciesdouglasfir: I(soildepth^2)	-0.097	0.049	-0.198	0.005				-0.156	-0.057
speciespine: I(soildepth^2)	-0.218	0.050	-0.326	-0.128				-0.263	-0.178

[(1)] species was dummy coded with beech being the reference level; the indicated test refers to the overall effect of species

[(2)] z-transformed to a mean of zero and a standard deviation (sd) of 1; mean and sd of the original variable were 167.5 and 121.8 cm, respectively.

Table S13 Estimates of the random effects part of the LMM with SRA as the response variable.

Groups	Effect [(1)]	Std.Dev.
pit	(Intercept)	0.002
	soil depth	<0.001
	I(soil depth^2)	0.024

[(1)] '(Intercept)' denotes a random intercept effect; others indicate random slopes effect. Species was dummy coded with beech being the reference level and then centered. Soil depth was z-transformed to a mean of 0 and a standard deviation of 1; mean and sd of the original variables were 167.5 and 121.8 cm.

Table S14 Estimates of the fixed effects part of the LMM with RAI as the response variable (estimates and standard errors, together with confidence limits, significance tests, and the range of estimates obtained when dropping levels of grouping factors one at a time).

	Estimate	Std. error	CL_{lower}	CL_{upper}	F	df	P	min	max
(Intercept)	-2.735	0.326	-3.332	-2.108				-2.881	-2.446
speciesoak [1]	-0.086	0.461	-1.001	0.758				-0.375	0.060
speciesdouglasfir	-0.802	0.461	-1.705	0.147				-1.257	-0.552
speciespine	-0.691	0.461	-1.563	0.158				-1.237	-0.330
soildepth [2]	-1.799	0.206	-2.196	-1.416				-1.895	-1.704
I(soildepth^2)	0.484	0.159	0.180	0.814				0.392	0.567
speciesoak: soildepth	0.120	0.292	-0.452	0.665				-0.014	0.331
speciesdouglasfir: soildepth	-0.419	0.292	-0.983	0.203				-0.530	-0.211
speciespine: soildepth	-0.049	0.293	-0.586	0.515				-0.364	0.285
speciesoak: I(soildepth^2)	0.127	0.225	-0.339	0.554	0.365	3, 8.5	0.780	-0.073	0.281
speciesdouglasfir: I(soildepth^2)	0.198	0.225	-0.262	0.605				0.072	0.392
speciespine: I(soildepth^2)	-0.043	0.228	-0.464	0.409				-0.199	0.148

[1] species was dummy coded with beech being the reference level; the indicated test refers to the overall effect of species

[2] z-transformed to a mean of zero and a standard deviation (sd) of 1; mean and sd of the original variable were 167.5 and 121.8 cm, respectively.

Table S15 Estimates of the random effects part of the LMM with RAI as the response variable.

Groups	Effect [1]	Std.Dev.
pit	(Intercept)	0.517
	soil depth	0.324
	I(soil depth^2)	0.213

[1] '(Intercept)' denotes a random intercept effect; others indicate random slopes effect. Species was dummy coded with beech being the reference level and then centered. Soil depth was z-transformed to a mean of 0 and a standard deviation of 1; mean and sd of the original variables were 167.5 and 121.8 cm.

Table S16 Estimates of the fixed effects part of the GLMM with proportion of vital ECM as the response variable (estimates and standard errors, together with confidence limits, significance tests, and the range of estimates obtained when dropping levels of grouping factors one at a time).

	Estimate	Std. error	CL_{lower}	CL_{upper}	x^2	df	P	min	max
(Intercept)	-2.225	0.149	-2.536	-1.943				-2.296	-2.171
speciesdouglasfir (1)	-0.125	0.213	-0.527	0.262				-0.26	-0.031
Speciesoak	1.788	0.212	1.390	2.218				1.618	1.942
soildepth (2)	-0.741	0.122	-0.997	-0.514				-0.907	-0.596
I(soildepth^2)	0.341	0.124	0.105	0.591				0.312	0.448
speciesdouglasfir: soildepth	0.216	0.174	-0.114	0.562				0.066	0.376
speciesoak: soildepth	0.774	0.173	0.441	1.135				0.628	0.943
speciesdouglasfir: I(soildepth^2)	0.295	0.178	-0.041	0.647	10	1	<0.001	0.087	0.431
speciesoak: I(soildepth^2)	-0.331	0.176	-0.697	0.025				-0.438	-0.27

(1) species was dummy coded with beech being the reference level; the indicated test refers to the overall effect of species

(2) z-transformed to a mean of zero and a standard deviation (sd) of 1; mean and sd of the original variable were 167.5 and 121.8 cm, respectively.

Table S17 Estimates of the random effects part of the GLMM with proportion of vital ECM as the response variable.

Groups	Effect (1)	Std.Dev.
pit	(Intercept)	0.628
	soil depth	0.124
	I(soil depth^2)	0.068

(1) '(Intercept)' denotes a random intercept effect; others indicate random slopes effect. Species was dummy coded with beech being the reference level and then centered. Soil depth was z-transformed to a mean of 0 and a standard deviation of 1; mean and sd of the original variables were 167.5 and 121.8 cm.

Fig. S1 Principal component analyses (PCA) visualizing root trait variation along the soil depth profile of four temperate tree species. a) PCA focused on core traits of the RES (SRL, mean diameter, root N and RTD), with dot colors indicating different species. Detailed PCA scores are provided in Table S18. ANOSIM test was significant ($p < 0.001$) and revealed an R value of 0.2145). b) PCA of all investigated trait variables, including SRL, mean diameter, Root N, RTD, SRA, and SRTA (specific root tip abundance) across all species. Detailed PCA scores are provided in Table S19. Dot colors represent different soil depth layers. PCA was carried out using the following functions and packages: prcomp, vegan (version 2.6-8) (Oksanen et al., 2024), ggplot2 (version 3.5.1) (Wickham, 2016), factoextra (version 1.0.7) (Kassambara and Mundt, 2020) and ggrepel (version 0.9.6) (Slowikowski, 2024).

a)

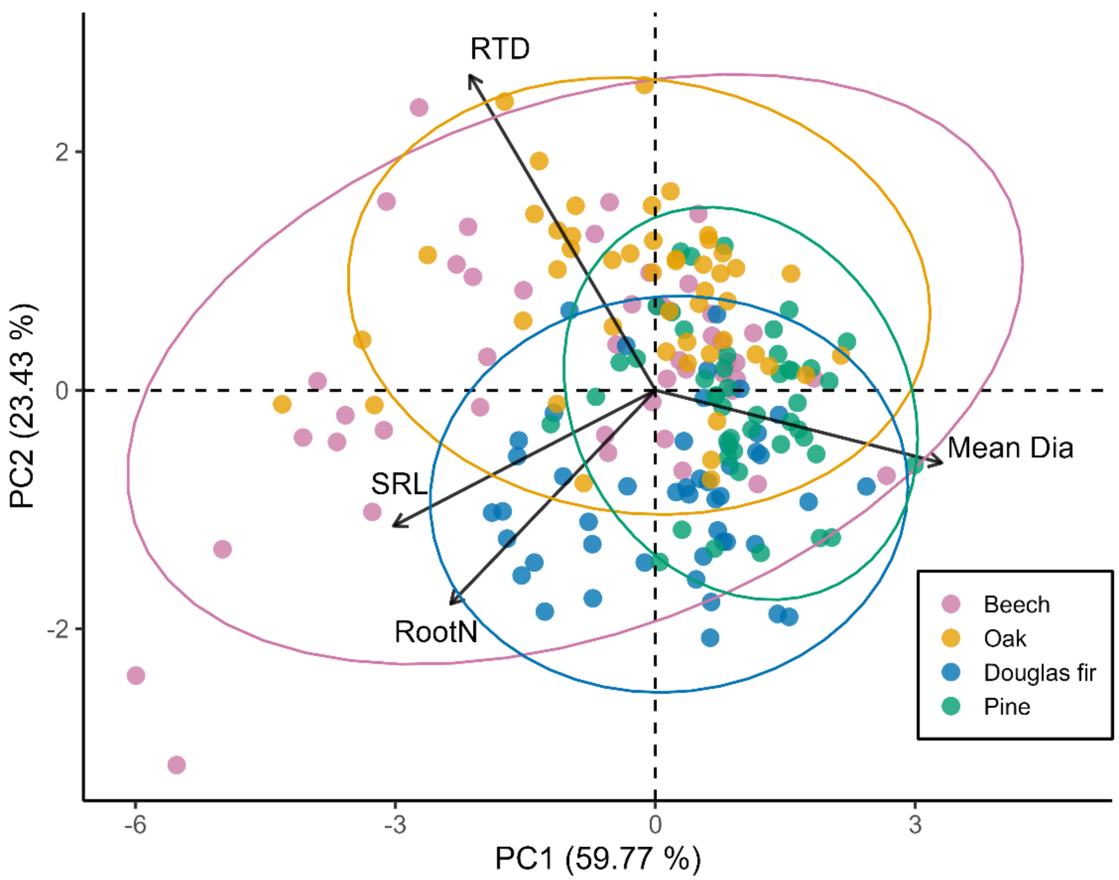

b)

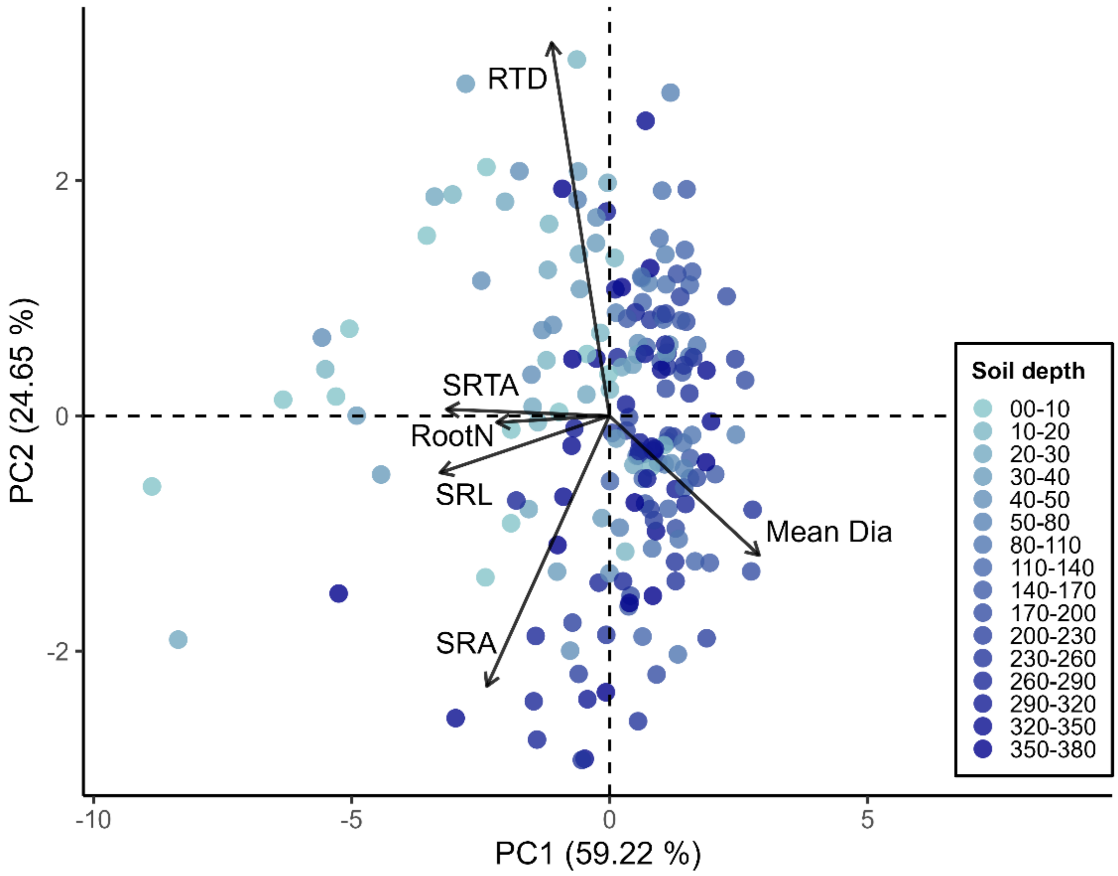

Table S18 Trait loadings of the principal component analysis of the core root economic traits; SRL, Mean Dia., root N and RTD (displayed in Fig. S1a). The proportion of variance explained is listed with each axis.

	PC1 (0.5977)	PC2 (0.2343)	PC3 (0.141)
RTD	-0.3898	0.7671	0.3542
SRL	-0.5496	-0.3305	-0.4951
Mean Dia.	0.6010	-0.1770	0.3018
Root N	-0.4299	-0.5205	0.7337

Table S19 Trait loadings of the principal component analysis of the root traits; SRL, Mean Dia., root N, RTD, SRA and SRTA (displayed in Fig. S1b). The proportion of variance explained is listed with each axis.

	PC1 (0.5922)	PC2 (0.2465)	PC3 (0.1113)
RTD	-0.1755	0.7696	-0.0422
SRL	-0.5134	-0.1172	-0.1690
SRA	-0.3719	-0.5577	-0.1716
Mean Dia.	0.4540	-0.2875	0.2398
SRTA	-0.4944	0.0134	-0.1057
Root N	-0.3418	-0.0135	0.9336

Fig. S2 Pearson correlation matrix with linear trends for the evaluated root and environmental traits: Log SRL (log-transformed specific root length), Mean Dia. (Mean diameter), Log root N (log-transformed root nitrogen), RTD (Root tissue density), Log soil C/N (log-transformed soil C/N), Log base pool (log-transformed base pool), Log AWC (log-transformed plant-available water capacity), BD (bulk density). Data points for each observed trait are displayed in a scatterplot, while the heatmap represents pairwise correlation coefficients, with values ranging from -1 to +1 to indicate the direction and strength of the relationships (negative to positive). The correlation matrix was generated using the corrmorant package (version 0.0.0 .9007; R. Link) and using the package ggplot2 (version 3.3.5; Wickham, 2016) within R version 4.3.0 (R Core Team, 2023).

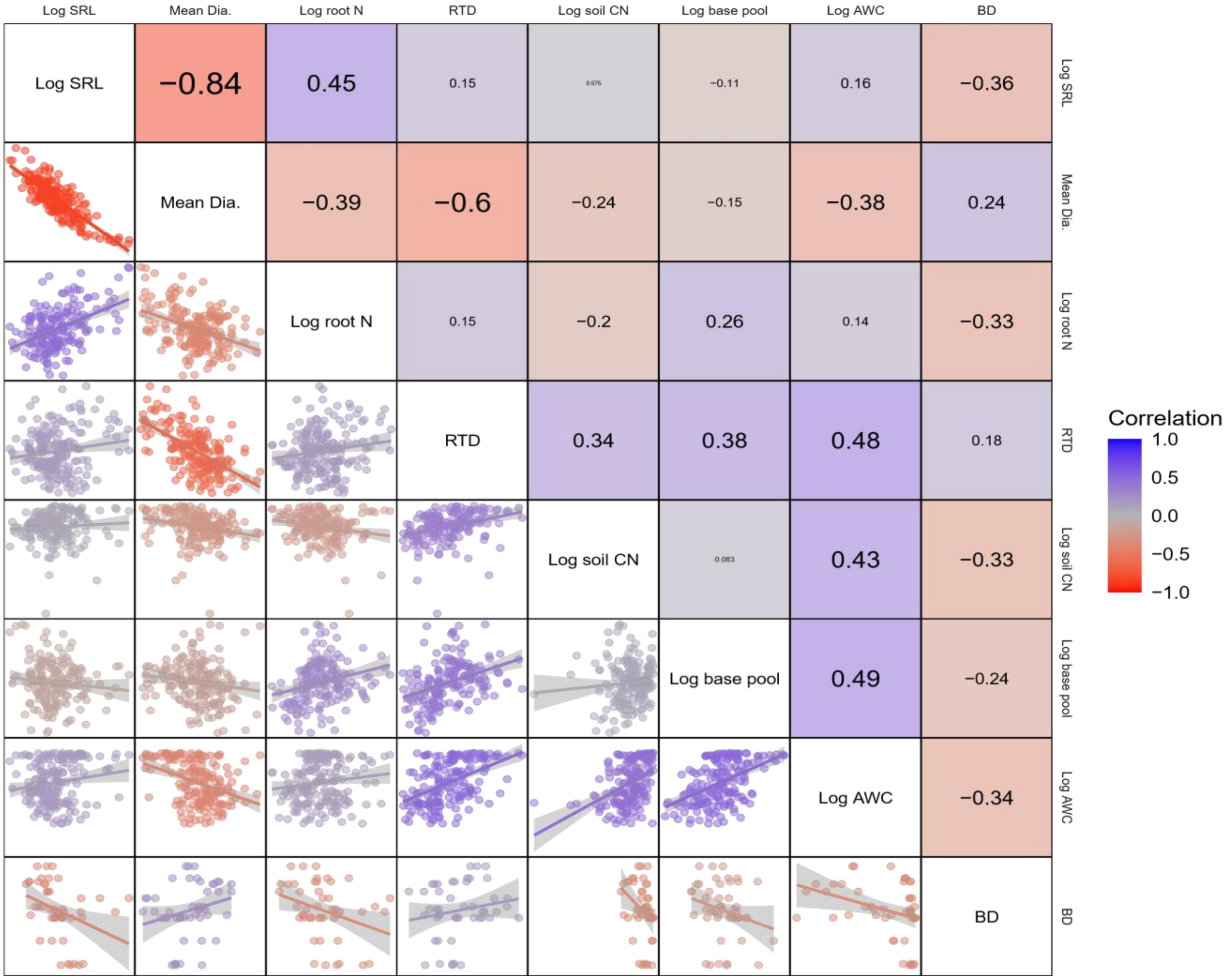

Fig. S3 Pearson correlation matrix with linear trends for more evaluated root and environmental traits: Log SRA (log-transformed specific root area), Log RAI (log-transformed root area index), Log SRTA (log transformed specific root tip abundance), proportion of vital ECM, Log soil C/N (log-transformed soil C/N), Log base pool (log-transformed base pool), Log AWC (log-transformed plant-available water capacity), BD (bulk density). Data points for each observed trait are displayed in a scatterplot, while the heatmap represents pairwise correlation coefficients, with values ranging from -1 to +1 to indicate the direction and strength of the relationships (negative to positive). The correlation matrix was generated using the corrmorant package (version 0.0.0 .9007; R. Link) and using the package ggplot2 (version 3.3.5; Wickham, 2016) within R version 4.3.0 (R Core Team, 2023).

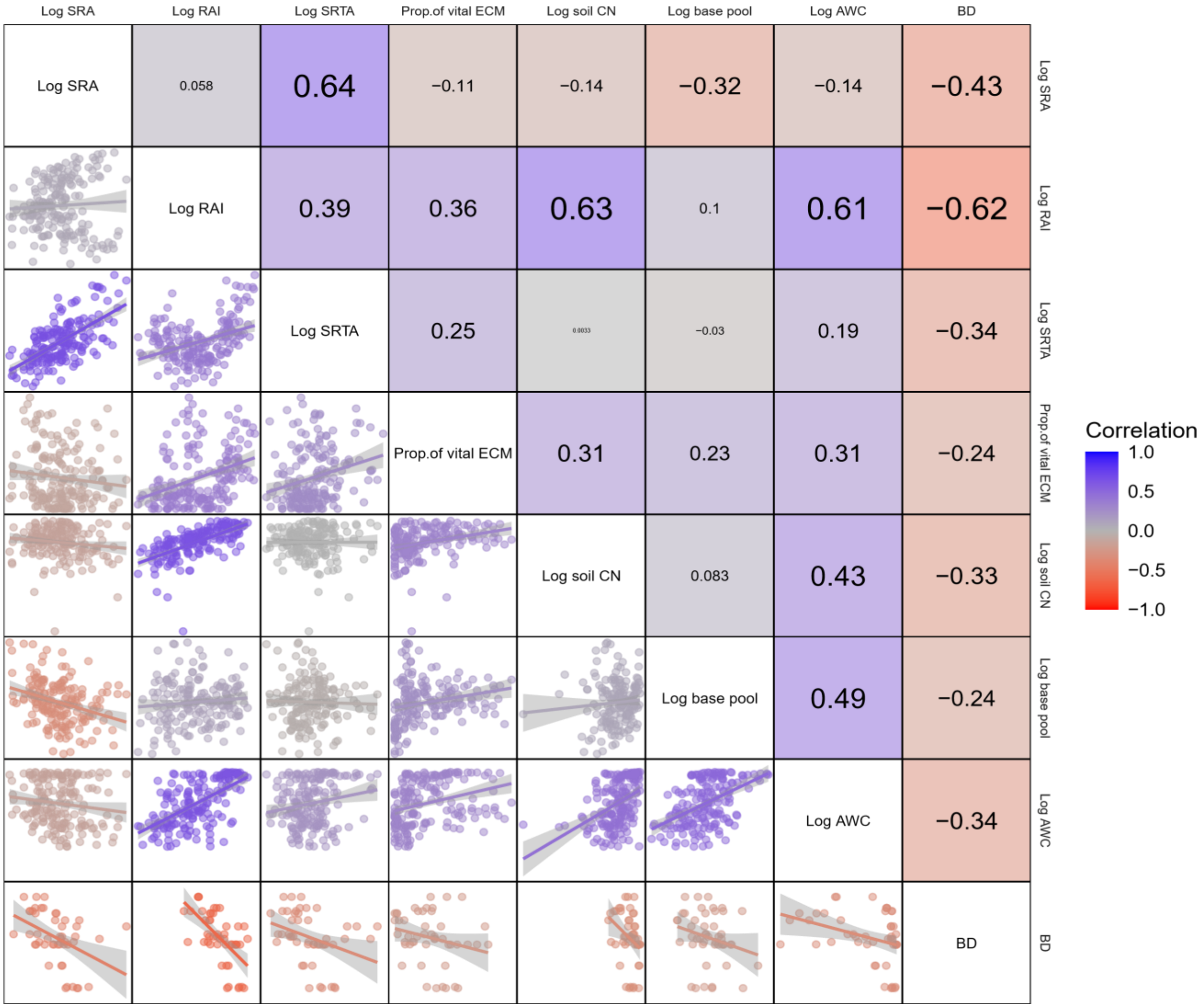

Fig. S4 Principal component analyses (PCA) visualizing variation of soil properties. a) PCA focused on soil C/N, soil N, AWC and base pool, with dot colors indicating different species. Detailed PCA scores are provided in Table S20. b) PCA of the same soil properties, but dot colors represent different soil depth layers.

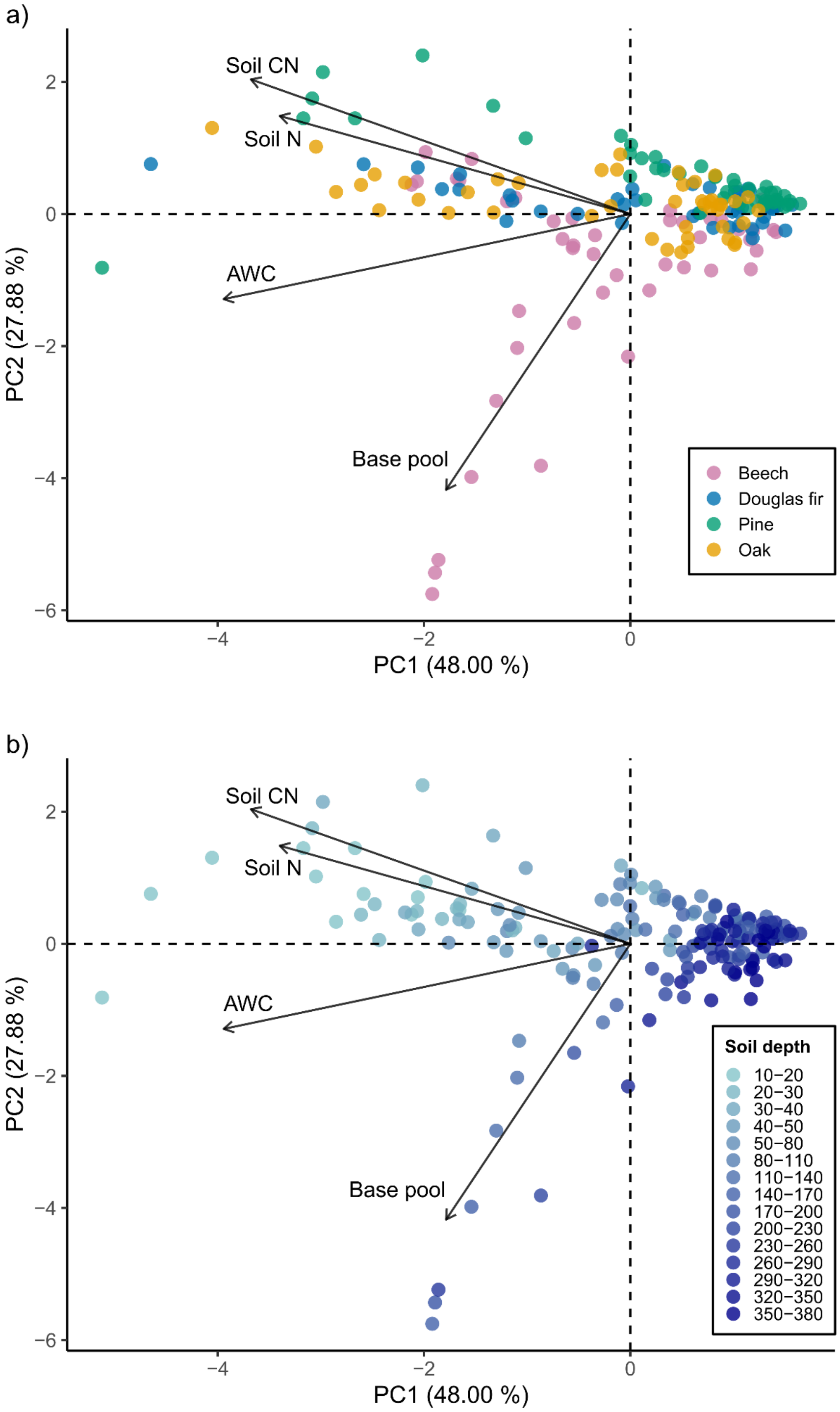

Table S20 Trait loadings of the principal component analysis of the soil properties: soil C/N, soil N, AWC and base pool (displayed in Fig. S4). The proportion of variance explained is listed with each axis.

	PC1 (0.480)	PC2 (0.279)	PC3 (0.155)
Soil C/N	-0.5557	0.4039	0.3963
Base pool	-0.2699	-0.8277	-0.2150
Soil N	-0.5135	0.2942	-0.7924
AWC	-0.5956	-0.2555	0.4108

References Supporting Information

Baayen, R. H. (2008) Analyzing linguistic data: A practical introduction to statistics using R, Cambridge, Cambridge University Press.
Barr, D. J., Levy, R., Scheepers, C. and Tily, H. J. (2013) 'Random effects structure for confirmatory hypothesis testing: Keep it maximal', Journal of Memory and Language, vol. 68, no. 3, pp. 255–278.
Bates, D., Mächler, M., Bolker, B. and Walker, S. (2015) 'Fitting Linear Mixed-Effects Models Using lme4', Journal of Statistical Software, vol. 67, no. 1, pp. 1–48.
Field, A. (2005) Discovering statistics using SPSS, London, Sage Publications.
Fox, J. and Weisberg, S. (2011) An R companion to applied regression, 2nd edn, Thousand Oaks CA, Sage.
Kassambara, A. and Mundt, F. (2020) factoextra: Extract and Visualize the Results of Multivariate Data Analyses: R Package Version 1.0.7. [Online]. Available at https://cran.r-project.org/package=factoextra (Accessed 21 April 2025).
Link, R. (2020) Flexible Correlation Matrices Based on 'ggplot2' corrmorant package [Online]. Available at https://github.com/r-link/corrmorant (Accessed 22 April 2025).
Matuschek, H., Kliegl, R., Vasishth, S., Baayen, H. and Bates, D. (2017) 'Balancing Type I error and power in linear mixed models', Journal of Memory and Language, vol. 94, pp. 305–315.
Nieuwenhuis, R., Grotenhuis, M. and Pelzer, B. (2012) 'influence.ME: Tools for Detecting Influential Data in Mixed Effects Models', The R Journal, vol. 4, no. 2, pp. 38–47.
Oksanen, J., Simpson, G. L., Blanchet, F. G., Kindt, R., Legendre, P., Minchin, P. R., O'Hara, R. B., Solymos, P., Stevens, M. H. H., Szoecs, E., Wagner, H., Barbour, M., Bedward, M., Bolker, B., Borcard, D., Carvalho, G., Chirico, M., Caceres, M. de, Durand, S., Evangelista, H. B. A., FitzJohn, R., Friendly, M., Furneaux, B., Hannigan, G., Hill, M. O., Lahti, L., McGlinn, D., Ouellette, M.-H., Ribeiro Cunha, E., Smith, T., Stier, A., Braak, C. J. ter, Weedon, J. and Borman, T. (2024) vegan: Community Ecology Package [Online]. Available at {https://CRAN.R-project.org/package=vegan}.
Quinn, G. P. and Keough, M. J. (2002) Experimental Designs and Data Analysis for Biologists, Cambridge, Cambridge University Press.
R Core Team (2023) R: A Language and Environment for Statistical Computing, Vienna, R Foundation for Statistical Computing, Available at https://www.r-project.org/.
RStudio Team (2023) RStudio: Integrated Development for R [Online], Boston, MA, Posit Software, PBC. Available at http://www.posit.co/.
Schielzeth, H. (2010) 'Simple means to improve the interpretability of regression coefficients', Methods in Ecology and Evolution, vol. 1, no. 2, pp. 103–113.
Schielzeth, H. and Forstmeier, W. (2009) 'Conclusions beyond support: overconfident estimates in mixed models', Behavioral Ecology, vol. 20, no. 2, pp. 416–420.
Slowikowski, K. (2024) 'ggrepel: Automatically Position Non-Overlapping Text Labels with 'ggplot2''.
Wickham, H. (2016) ggplot2: Elegant graphics for data analysis, 2nd edn, Berlin, Heidelberg, Springer.
